# TBX2-positive cells represent a multi-potent mesenchymal progenitor pool in the developing lung

**DOI:** 10.1186/s12931-019-1264-y

**Published:** 2019-12-23

**Authors:** Irina Wojahn, Timo H. Lüdtke, Vincent M. Christoffels, Mark-Oliver Trowe, Andreas Kispert

**Affiliations:** 1 Institut für Molekularbiologie, Medizinische Hochschule Hannover, Hannover, Germany; 2 Department of Anatomy, Embryology and Physiology, Academic Medical Center, University of Amsterdam, Amsterdam, Netherlands

**Keywords:** Tbx2, Lineage tracing, Pulmonary mesenchyme, Smooth muscle cells, Lung development

## Abstract

**Background:**

In the embryonic mammalian lung, mesenchymal cells act both as a signaling center for epithelial proliferation, differentiation and morphogenesis as well as a source for a multitude of differentiated cell types that support the structure of the developing and mature organ. Whether the embryonic pulmonary mesenchyme is a homogenous precursor pool and how it diversifies into different cell lineages is poorly understood. We have previously shown that the T-box transcription factor gene *Tbx2* is expressed in the pulmonary mesenchyme of the developing murine lung and is required therein to maintain branching morphogenesis.

**Methods:**

We determined Tbx2/TBX2 expression in the developing murine lung by in situ hybridization and immunofluorescence analyses. We used a genetic lineage tracing approach with a *Cre* line under the control of endogenous *Tbx2* control elements (*Tbx2*^*cre*^), and the *R26*^*mTmG*^ reporter line to trace TBX2-positive cells in the murine lung. We determined the fate of the TBX2 lineage by co-immunofluorescence analysis of the GFP reporter and differentiation markers in normal murine lungs and in lungs lacking or overexpressing TBX2 in the pulmonary mesenchyme.

**Results:**

We show that TBX2 is strongly expressed in mesenchymal progenitors in the developing murine lung. In differentiated smooth muscle cells and in fibroblasts, expression of TBX2 is still widespread but strongly reduced. In mesothelial and endothelial cells expression is more variable and scattered. All fetal smooth muscle cells, endothelial cells and fibroblasts derive from TBX2^+^ progenitors, whereas half of the mesothelial cells have a different descent. The fate of TBX2-expressing cells is not changed in *Tbx2*-deficient and in *TBX2-*constitutively overexpressing mice but the distribution and abundance of endothelial and smooth muscle cells is changed in the overexpression condition.

**Conclusion:**

The fate of pulmonary mesenchymal progenitors is largely independent of TBX2. Nevertheless, a successive and precisely timed downregulation of TBX2 is necessary to allow proper differentiation and functionality of bronchial smooth muscle cells and to limit endothelial differentiation. Our work suggests expression of TBX2 in an early pulmonary mesenchymal progenitor and supports a role of TBX2 in maintaining the precursor state of these cells.

## Background

The primary function of the lung, the exchange of oxygen in the air with carbon dioxide in the vascular system, is supported by a multitude of differentiated cell types in a highly organized tissue architecture. Predominant are epithelial cells that line both the conducting airways of the trachea and the bronchial tree as well as the distal gas-exchange units, the alveoli. According to their position, epithelial cells are diversified to support the exclusion of solid particles and fight microorganisms on the one hand, and to allow intimate association and gas exchange with the highly elaborated vascular system on the other hand [1, 2]. Mesenchymal cells line the respiratory epithelium and are similarly specialized along the proximal to distal axis of the lung. From the trachea down to the bronchi they form cartilaginous rings and smooth muscle cells (SMCs) in an alternating fashion to stabilize the airways. In the bronchial tree SMCs are highly abundant, while only a sparse population of fibroblasts resides in the alveolar interstitium. In the entire organ, mesenchymal cells associate as pericytes and SMCs with the endothelial network [3]. Finally, a layer of mesothelium, the visceral pleura, covers the outside of the organ possibly to synthesize lubricating factors and help in defense of pathogens [4].

Mesenchymal cells provide structural support to the respiratory epithelium and the vessels under homeostatic conditions but also play an indispensable instructive role at all steps of pulmonary epithelial development in embryogenesis (for reviews see [5, 6]. At the onset of lung development, in the mouse around embryonic day (E)9.0, mesenchyme surrounding the ventral anterior foregut endoderm acts as a critical source of signals that specifies the pulmonary epithelium [7] and induces its evagination and division into the first two lung buds. Throughout the extended pseudoglandular stage, which in the mouse ends around E16.5, mesenchymal signals direct the elongation and branching of the lung buds into the bronchial tree [8, 9], and account for their correct proximal-distal patterning and differentiation [10]. Finally, the mesenchyme is important for septation of the distal air-sacs, the alveoli, in the canalicular and saccular phases from E16.5 onwards [11, 12].

During this developmental time-line mesenchymal progenitors residing at the distal lung buds differentiate in a temporally and spatially specific manner into a multitude of cell types starting proximally with airway and vascular SMCs, pericytes, and airway cartilage cells, and ending with distal alveolar lipo- and myofibroblasts [3]. Mesenchymal and epithelial development is also supported by the embryonic mesothelium which forms shortly after specification of the lung bud. The mesothelium provides crucial signals to maintain mesenchymal proliferation and may act as a minor cell source for the pulmonary mesenchyme [13–15] (for recent reviews on lung development and structure see [16, 17]).

Despite its important developmental function, our knowledge of mesenchymal (and mesothelial) differentiation clearly lags behind that of the epithelium. We have recently characterized TBX2, a member of the T-box family of transcription factors, as a crucial mesenchymal factor for embryonic lung development. Expression of *Tbx2* occurs in the pulmonary mesenchyme from E9.5 to at least E18.5. Loss of *Tbx2* function leads to reduced mesenchymal proliferation, but also affects in a non cell-autonomous fashion proliferation of the distal epithelium and branching morphogenesis resulting in lung hypoplasia from E14.5 onwards. Epithelial patterning is not affected upon loss of *Tbx2* in the mesenchyme, but the number of alveolar epithelial cells type I is mildly reduced at E18.5. Constitutive TBX2 expression in mature lungs results in mesenchymal hyperproliferation, but does not affect branching morphogenesis or epithelial differentiation [18]. Molecular analysis showed that TBX2 maintains mesenchymal proliferation by repressing *Cdkn1a* (p21) and *Cdkn1b* (p27), two members of the Cip/Kip family of cell cycle inhibitor genes [18], and independently, by maintaining pro-proliferative WNT signaling through repression of WNT antagonist genes *Frzb* and *Shisa3* [19].

Here, we further characterize the pool of TBX2 positive cells in the developing lung, and determine its contribution to differentiated mesenchymal cells types in normal development but also under conditions of mesenchymal loss and gain of *Tbx2.* We provide evidence that TBX2 not only marks a multipotent precursor population in the pulmonary mesenchyme and maintains its undifferentiated state, but is also essential for proper SMC functionality.

## Materials and methods

### Mouse strains and genotyping

*Tbx2*^*tm1.1(cre)Vmc*^ (synonym: *Tbx2*^*cre*^) [20], *Tbx2*^*tm2.2Vmc*^ (synonym: *Tbx2*^*fl*^) [21], *Gt (ROSA)26*^*Sortm4(ACTB-tdTomato,-EGFP)Luo/J*^ (synonym: *R26*^*mTmG*^) [22] and *Hprt*^*tm2(CAG-TBX2,-EGFP)Akis*^ (synonym*: Hprt*^*TBX*2^) [23] mice were maintained on an NMRI outbred background. Embryos for phenotype analysis were derived from matings of *Tbx2*^*cre/+*^ males with *Tbx2*^*fl/fl*^*;R26*^*mTmG/mTmG*^, *Hprt*^*TBX2/TBX2*^ or *R26*^*mTmG/mTmG*^ females. For timed pregnancies, vaginal plugs were checked on the morning after mating and noon was taken as embryonic day (E) 0.5. Pregnant females were sacrificed by cervical dislocation. Embryos were isolated in PBS, decapitated, rinsed and fixed in 4% paraformaldehyde (PFA)/PBS overnight and stored in 100% methanol at − 20 °C until use. Genotypes of embryos were assigned by epifluorescence analysis of GFP expression from the reporter allele or from the *Hprt* allele.

All animal work conducted for this study was performed according to European and German legislation. The breeding, handling and sacrifice of mice for embryo isolation was approved by the Niedersächsisches Landesamt für Verbraucherschutz und Lebensmittelsicherheit (Permit Number: AZ33.12–42,502–04-13/1356).

### Organ culture

Lung rudiments of E12.5 embryos were explanted on 0.4 μm polyester membrane Transwell supports (#3450, Corning Inc., Lowell, MA, USA) and cultivated at the air-liquid interface for 36 h, 6 days or 8 days at 37 °C and 5% CO_2_ in RPMI medium (#61870044, ThermoFisher Scientific, Waltham, MA, USA) supplemented with 10% FCS (#S0115, Biochrom, Berlin, Germany), 100 units/ml Penicillin/100 μg/ml Streptomycin (#15140 122, ThermoFisher Scientific).

To record contractility in cultures, videos of 2 min length were taken 12 h, 18 h, 24 h and 36 h after explantation. Only lungs of comparable developmental stage as judged by the number of branching endpoints were included in this assay. Contraction intensity was measured by computational Fiji Multi-Kymograph analysis (www.imagej.net) [24]. To compare these intensities over a full contraction wave, we determined the area below the intensity curves. Results of both were statistically evaluated by two-tailed Student’s t-test and considered significant (*P* < 0.05), highly significant (*P* < 0.01), or extremely significant (*P* < 0.001).

### Immunofluorescence

Detection of antigens was performed on 5 μm paraffin sections. Endogenous peroxidases were blocked by incubation in 6% H_2_O_2_ for 20 min. For antigen retrieval either 0.05% Triton X-100 (PDGFRA/B) or citrate-based heat unmasking (all others) was employed. The following primary antibodies were used: anti-ALDH1A2 (1:200; #ab96060, Abcam plc, Cambridge, UK), anti-CDH1 (1:500; a kind gift of Rolf Kemler, MPI Freiburg), anti-EMCN (1:2; a kind gift of Prof. Dietmar Vestweber, MPI Münster), mouse-anti-GFP (1:50, 1:200; #11814460001, Roche, Basel, Switzerland), rabbit-anti-GFP (1:100; #ab290, Abcam), anti-KDR (1:50, 1:200; #BAF644, R&D Systems, Minneapolis, MN, USA), anti-PDGRFA (1:200; #AF1062-SP, R&D Systems), anti-PDGFRB (1:200; #AF1042-SP, R&D Systems), anti-POSTN (1:200; #ab14041, Abcam), anti-S100A4 (1:200; #ab27957–250, Abcam), anti-ACTA2 (1:200; #A5228, Sigma-Aldrich, St. Louis, MO, USA), anti-TAGLN (1:400; #ab14106–100, Abcam), anti-TBX2 (1:200, 1:2000; #07–318, Merck Millipore, Darmstadt, Germany), anti-TBX2 (1:200; #sc-514,291 X, Santa Cruz Biotechnology, Inc., Dallas, TX, USA), anti-TBX3 (1:50; #sc-31,656, Santa Cruz), anti-WT1 (1:500; #CA1026, Calbiochem, San Diego, CA, USA)*.* Primary antibodies were detected by directly labeled fluorescence- or biotin-conjugated secondary antibodies (1:200; Invitrogen, Carlsbad, CA, USA; Dianova, Hamburg, Germany; Jackson ImmunoResearch, Cambridgeshire, UK). Signal amplification was performed with the Tyramide Signal Amplification (TSA) system (NEL702001KT, PerkinElmer, Waltham, MA, USA) according to the manufacturer’s instruction. Nuclei were stained with 4′,6-diamidino-2-phenylindole (DAPI, # 6335.1, Carl Roth, Karlsruhe, Germany). To exclude unspecific binding of secondary or tertiary antibodies, we performed as a control immunofluorescence stainings without primary antibody and if required without primary and secondary antibodies (Additional file 1: Figure S1).

### Quantification of immunofluorescence staining

We used Fiji freeware (www.imagej.net) to quantify the relative expression of TBX2 and of the lineage reporter GFP at different developmental time-points in the entire lung mesenchyme (10.5, E12.5), in the mesenchyme of the right lung lobe (E14.5, E16.5) and in regions of specific cell types (E14.5) in a semi-quantitative manner.

The mesenchymal compartment was defined by DAPI-signal based histology, whereas cell-type specific areas were defined by marker gene expression. The specific immunofluorescence signals of each single color-channel picture were converted into black pixels, while signal negative areas of the picture were displayed in white. The area of black pixels was measured. The relative area of DAPI or of a specific marker was set to 100%. Within this area, the proportion representing TBX2 or GFP expression was calculated as the ratio of TBX2 (or GFP) area to DAPI (or marker) area and expressed in %. Measurements were performed for at least three individuals (exception: *n* = 2 for TBX2 expression in PDGFRA^+^ and PDGFRB^+^ cells) and data were expressed as means±SD. Differences in GFP expression of control and *Tbx2*-deficient mice were compared and considered significant with *p ≤ 0.05, ** *p* ≤ 0.01, *** *p* ≤ 0.005, using two-tailed Student’s t-test. The complete data set is provided in Additional file 2: Table S1.

### RNA in situ hybridization analysis

In situ hybridization were performed on 5-μm or 10-μm paraffin sections as described [25]. For each marker, at least three independent specimens were analyzed.

### Documentation

Overviews of sectioned lungs were documented either with a DM5000 or DM6000 microscope (Leica Camera, Wetzlar, Germany) equipped with a Leica DFC300FX or Leica DFC350FX digital camera, respectively. For higher magnifications confocal microscopy was performed using a Leica DM IRB with a TCS SP2 AOBS scan head. Organ cultures were photographed with the DM6000 microscope, a Leica M420 microscope with a Fujix digital camera HC-300Z (Fujifilm Holdings, Minato/Tokyo, Japan) or a Leica MZFLIII with a Leica DFC420C digital camera. Images were processed and analyzed in Adobe Photoshop CS5 (Adobe, San Jose, CA, USA).

## Results

### TBX2 is expressed in a variety of cells excluding the airway epithelium during lung development

**Fig. 1:**
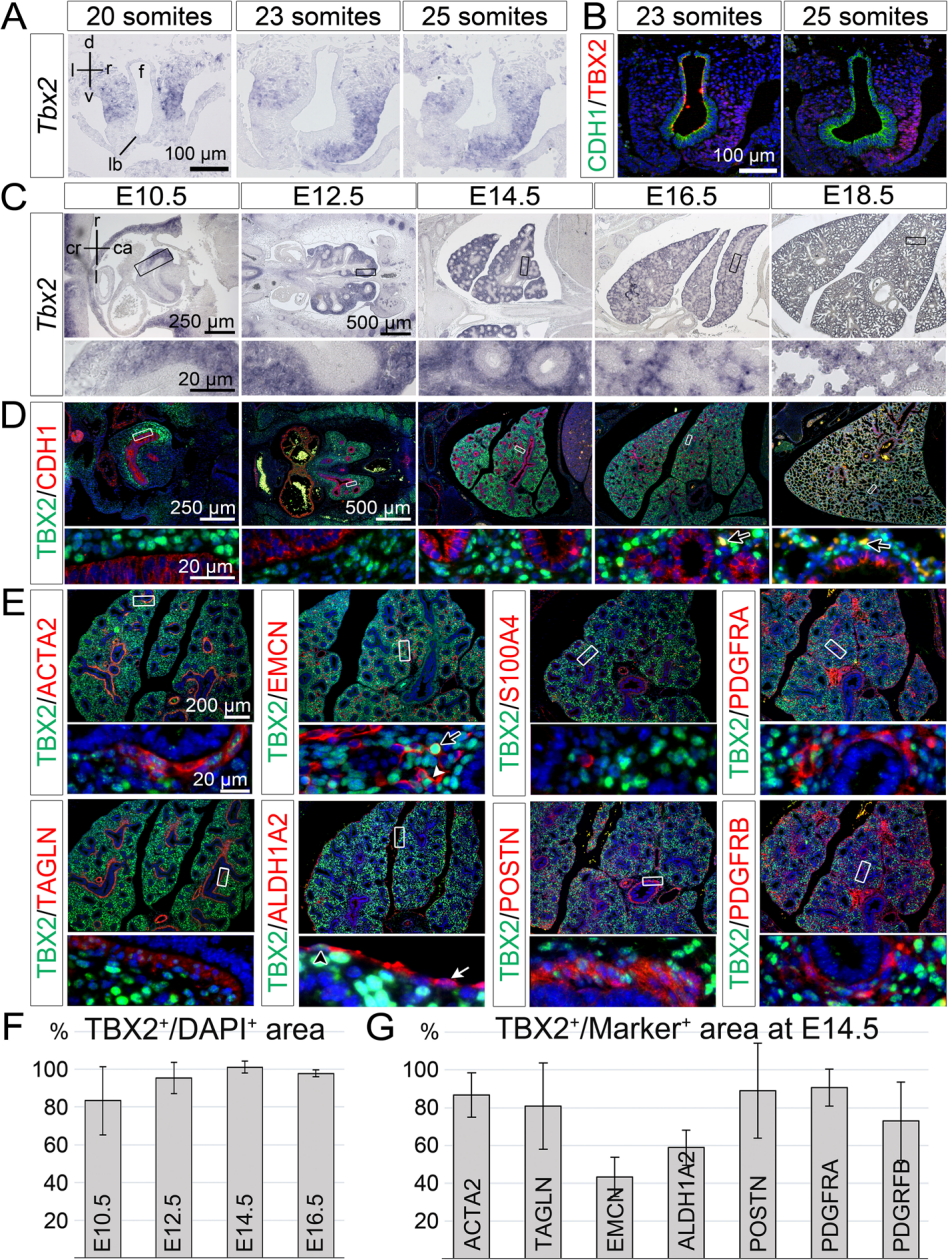
*Tbx2*/TBX2 is predominantly expressed in mesenchymal progenitors in the developing lung. (**a**, **b**, **c**, **d**) In situ hybridization analysis of *Tbx2* expression (**a**, **c**), and double immunofluorescence analysis of expression of TBX2 with the epithelial marker CDH1 (**b**, **d**) on transversal (**a**, **b**) and frontal (**c**, **d**) sections of the lung at different stages of wildtype mouse embryos. (**e**) Double immunofluorescence analysis of expression of TBX2 and of marker proteins for SMCs (TAGLN, ACTA2), the endothelium (EMCN), the mesothelium of the visceral pleura (ALDH1A) and different types of fibroblasts and ECM (POSTN, S100A4, PDGFRA, PDGFRB) on frontal sections of E14.5 wildtype lungs. (**f**) Quantification of mesenchymal TBX2 expression in the whole lung (E10.5 and E12.5) and in the right lung lobe (E14.5 and E16.5). Average values: E10.5: 83% ± 18%; E12.5: 95% ± 8%; E14.5: 101% ± 3%, E16.5: 98% ± 2%. (**g**) Quantification of cell-type specific TBX2 expression at E14.5. Average values: ACTA2^+^: 87% ± 12%; TAGLN^+^: 81% ± 23%; EMCN^+^: 43% ± 10%; ALDH1A2^+^: 59% ± 9%; POSTN^+^: 89% ± 25%; PDGFRA^+^: 91% ± 10%; PDGFRB^+^: 73% ± 21%. Complete data set is provided in Table S1. Values above 100% result from technical artefacts. Stages, probes and antigens are as indicated. Nuclei were counter stained with DAPI in immunofluorescence stainings. Insets of overview images are magnified in the row below. ca: caudal; cr: cranial; d: dorsal; f: foregut; l: left; lb.: lung bud; r: right; v: ventral. Black arrow: indicates a blood auto-fluorescent cell; white arrow: TBX2 negative mesothelial cell; white arrowhead: TBX2^+^ endothelial cell; black arrowhead: TBX2^+^ mesothelial cell

To define the spatial and temporal expression of *Tbx2* mRNA and TBX2 protein during murine lung development in greater detail as previously reported [18], we performed in situ hybridization and (co-)immunofluorescence analysis on lung sections of different developmental stages (Fig. 1). At E9.0 to E9.5, we visualized the lung bud as a *Nkx2.1*^*+*^ epithelium ventral to the foregut (Additional file 1: Figure S2). In embryos with 20 somites, *Tbx2* mRNA was restricted to the lateral foregut mesenchyme. At the 23-somite stage, both *Tbx2* mRNA and TBX2 protein expression expanded into the mesenchyme on the right side of the lung bud (Fig. 1A and B). At the 25-somite stage, *Tbx2*/TBX2 expression was increased in this region (Fig. 1a and b, Additional file 1: Figure S2A). From E10.5 to E16.5, *Tbx2* mRNA was robustly detected in the entire pulmonary mesenchyme, i.e. both in the undifferentiated mesenchyme surrounding the distal tip regions and more weakly in proximal regions where differentiated cell types reside. At E18.5, *Tbx2* expression was strongly decreased. The epithelium lacked *Tbx2* expression at all stages (Fig. 1a and c).

Double immunofluorescence analysis with the epithelial marker cadherin 1 (CDH1) confirmed complete absence of TBX2 protein from the airway epithelium. In the pulmonary mesenchyme, TBX2 expression was strongest in cells surrounding the distal epithelial lung buds. There as well as in more proximal regions some cells lacked TBX2 or expressed low levels only (Fig. 1d). To investigate whether this variable expression reflects a cell-type specific restriction, we performed double immunofluorescence stainings of TBX2 and markers of various differentiated cell-types that reside outside the airway epithelium (Fig. 1e). We performed this analysis at E14.5 when these cell-types are established and easy to visualize. TBX2 expression was found at low levels in actin, alpha 2, smooth muscle, aorta positive (ACTA2^+^) and transgelin positive (TAGLN^+^) bronchial SMCs and in some scattered endomucin positive (EMCN^+^) endothelial cells. Similarly, the mesothelial lining of the visceral pleura, which is marked by aldehyde dehydrogenase family 1, subfamily A2 (ALDH1A2) expression [26], contained TBX2^+^ cells. Fibroblasts constitute a heterogeneous, poorly characterized mesenchymal cell type in the lung. Some interstitial fibroblasts are marked by the expression of the S100 calcium binding protein A4 (S100A4) [27–29]. We did not find expression of this marker in TBX2^+^ cells. However, weak TBX2 expression was found in association with cells expressing periostin (POSTN), an extracellular matrix protein produced by fibroblasts surrounding the main bronchi at this stage [30], in cells expressing platelet derived growth factor receptor, alpha polypeptide (PDGFRA), a marker for (myo-)fibroblasts and SMC precursors [11, 12] and in cells positive for platelet derived growth factor receptor, beta polypeptide (PDGFRB), a marker for vascular SMC precursors and pericytes [31].

Quantification of TBX2 expression by Fiji-based measurement of the immunofluorescent signals confirmed TBX2 expression in most (E10.5) and almost all mesenchymal cells of the developing lung (E12.5, E14.5, E16.5) (Fig. 1f, Additional file 2: Table S1), and revealed that low level expression of TBX2 at E14.5 was detected in 40% of EMCN^+^ endothelial cells, 60% of ALDH1A2^+^ mesothelial cells and over 80% of ACTA2^+^, TAGLN^+^ SMCs and POSTN^+^, PDGFRA^+^ or PDGFRB^+^ (myo-)fibroblasts (Fig. 1g, Additional file 2: Table S1). Thus, TBX2 is strongly expressed in mesenchymal precursors, and persists at lower levels and to various degrees in differentiated cell-types including SMCs, pericytes and (myo-)fibroblasts, endothelial and mesothelial cells at this stage.

### Fibroblasts, endothelial, mesothelial and SM cells derive from a TBX2^+^ precursor population

**Fig. 2:**
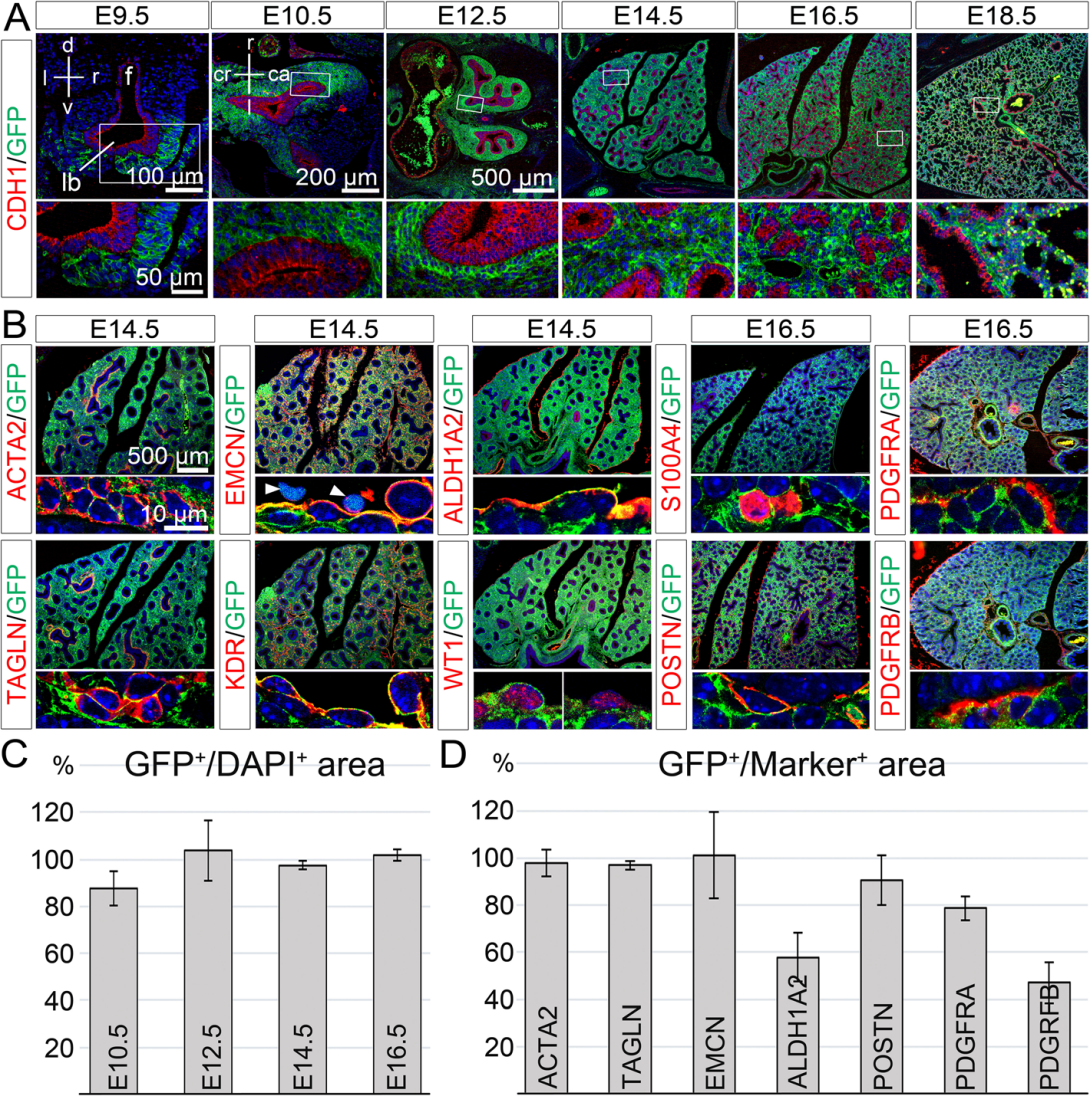
TBX2^+^ cells contribute to fibroblasts, endothelial, mesothelial and SM cells in the developing lung. (**a**) Double immunofluorescence analysis of the lineage marker GFP and the epithelial marker CDH1 on transversal (E9.5) and frontal (E10.5 and older) sections of *Tbx2*^*cre/+*^*;R26*^*mTmG/+*^ lungs. (**b**) Double immunofluorescence of the lineage marker GFP and marker proteins of SMCs (ACTA2, TAGLN), the endothelium (EMCN, KDR), the visceral pleura (ALDH1A2, WT1), different types of fibroblasts and ECM (POSTN, S100A4, PDGFRA, PDGFRB) on frontal lung sections of *Tbx2*^*cre/+*^*;R26*^*mTmG/+*^ embryos at representative stages. (**c**) Quantification of GFP signal reflecting the lineage contribution to the mesenchyme of the whole lung (E10.5 and E12.5) and the right lung lobe (E14.5, E16.5). Average values: E10.5: 88% ± 7%; E12.5: 103% ± 13%; E14.5: 98 ± 2%, E16.5: 102% ± 2%. (**d**) Quantification of GFP expression in specific cell-types at E14.5 and E16.5. Average values: ACTA2^+^: 98% ± 6%; TAGLN^+^: 97% ± 2%; EMCN^+^: 101 ± 18%; ALDH1A2^+^: 58% ± 10%; POSTN^+^: 91 ± 11%; PDGFRA^+^: 79% ± 5%; PDGFRB^+^: 47% ± 9%. The complete data set is provided in Table S1. Values above 100% are technical artefacts. Antigens are color-coded, stages are as indicated. Nuclei were counterstained with DAPI. Insets or selected regions of overview images are magnified in the row below. ca: caudal; cr: cranial; d: dorsal; f: foregut; l: left; lb.: lung bud; r: right; v: ventral. Arrowhead: indicates an auto-fluorescent cell

Since some mesenchymal (progenitor) cells surrounding the distal lung buds and most differentiated pulmonary cells lacked TBX2 or expressed only low levels, we questioned whether these cells are descendants of cells initially positive for the protein. To test this hypothesis, we used a genetic lineage tracing approach with a *Cre* line under the control of endogenous *Tbx2* control elements (*Tbx2*^*cre*^) [20], and the *R26*^*mTmG*^ reporter line which switches from membrane-bound RFP to membrane bound GFP expression upon *Cre-*mediated recombination [22]. We performed co-stainings of the lineage marker GFP with CDH1 during development (Fig. 2a), and of GFP with differentiation markers at E14.5 and E16.5 (Fig. 2b) on lung sections of *Tbx2*^*cre/+*^*;R26*^*mTmG/+*^ embryos, and quantified the signals to judge the overall contribution of TBX2^+^ cells to the epithelial and mesenchymal compartment (Fig. 2c, Additional file 2: Table S1) and to differentiated cell-types in the pulmonary mesenchyme (Fig. 2d, Additional file 2: Table S1).

GFP^+^ cells were found in a scattered fashion in the pulmonary mesenchyme at E9.5 (Fig. 2a). At E10.5, the contribution of TBX2^+^ cells to cell types outside the airway epithelium was 88% and increased to almost 100% at E12.5, E14.5 and E16.5 (Fig. 2a and c, Additional file 2: Table S1). All ACTA2^+^ and TAGLN^+^ SMCs were positive for the lineage marker GFP at E14.5, as were EMCN- and kinase insert domain protein receptor (KDR) [32, 33] positive endothelial cells (Fig. 2b and d, Additional file 2: Table S1). We also observed co-expression of ALDH1A2 (58%) and wilms tumor 1 homolog (WT1) [34], two mesothelial markers, with GFP. Moreover, most if not all S100A4^+^ cells (not quantifiable by Fiji-based tools), 91% of POSTN-, 79% of PDGFRA- and 47% of PDGFRB-expressing cells were positive for GFP expression at E16.5 (Fig. 2b and d, Additional file 2: Table S1). Together this analysis shows that SMCs, endothelial cells and fibroblasts of the fetal lung derive almost completely, mesothelial cells to about 50% from cells positive for TBX2 expression.

### Lineage contribution of TBX2^+^ cells is not changed upon loss or gain of TBX2

Loss of *Tbx2* in the pulmonary mesenchyme leads to hypoplasia whereas overexpression results in tissue thickening and organ overgrowth possibly by altering the balance between progenitor proliferation and differentiation [18, 19]. To determine whether these manipulations of TBX2 expression affect the lineage diversification of TBX2^+^ cells, we performed cell fate analysis in lungs of mice with conditional loss or gain of *Tbx2* expression in the pulmonary mesenchyme.

**Fig. 3:**
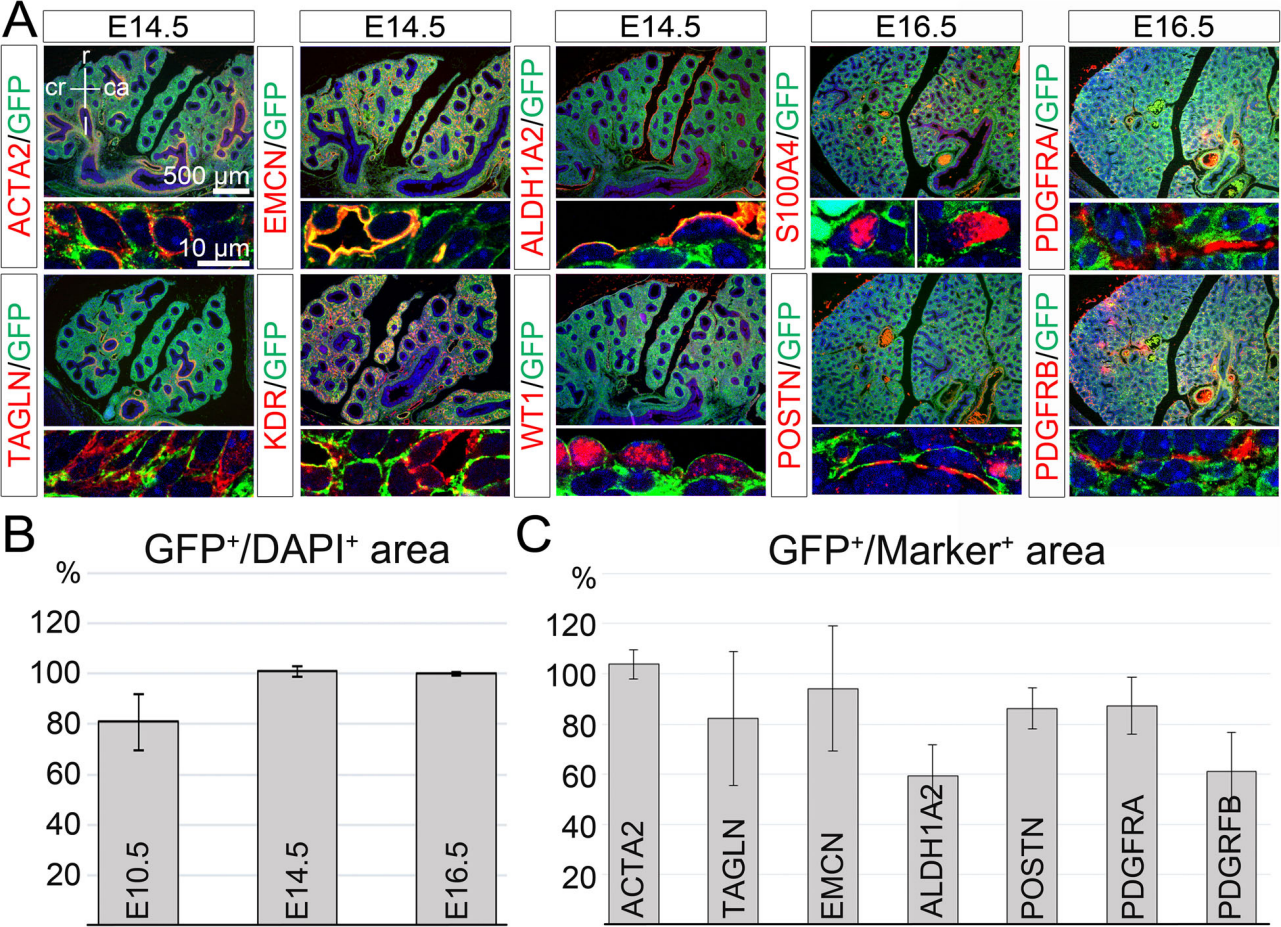
*Tbx2-*deficiency does not alter the fate of TBX2^+^ cells in the developing lung. (**a**) Double immunofluorescence analysis of expression of the lineage marker GFP with SMC proteins (ACTA2, TAGLN), and with markers of the endothelium (EMCN, KDR), the visceral pleura (ALDH1A2, WT1), different types of fibroblasts (S100A4, PDGFRA, PDGFRB) and the ECM (POSTN) on frontal lung sections of *Tbx2*-deficient (*Tbx2*^*cre/fl*^*;R26*^*mTmG/+*^) embryos at representative stages. (**b**) Quantification of the TBX2 lineage contribution reflected by GFP signal to the mesenchyme of the whole lung (E10.5) and the right lung lobe (E14.5 and E16.5) of *Tbx2*-deficient lungs. Average values: E10.5: 81 ± 11%; E14.5: 101% ± 2%; E16.5: 100% ± 1%. (**c**) Quantification of GFP signal in specific cell types (E14.5 and E16.5) upon *Tbx2* deletion. Average values: ACTA2^+^: 104% ± 6%; TAGLN^+^: 82% ± 27%; EMCN^+^: 94% ± 25%; ALDH1A2^+^: 60% ± 12%; POSTN^+^: 86% ± 8%; PDGFRA^+^: 87 ± 11%; PDGFRB^+^: 85% ± 31%. Complete data set is provided in Table S1. Values above 100 result from technical artefacts. Antigens are color-coded, stages are as indicated. Nuclei were counterstained with DAPI. Selected regions of overview images are magnified in the row below. ca: caudal; cr: cranial; l: left; r: right.

For this purpose, we combined the *Tbx2*^*cre*^ allele with a *Tbx2*^*floxed*^ allele [21] and the *R26*^*mTmG*^ reporter line [22]. Immunofluorescence analysis on lung sections of these *Tbx2*^*cre/fl*^;*R26*^*mTmG/+*^ embryos at E9.5, E10.5 and E11.5 confirmed the absence of TBX2 protein in the entire pulmonary mesenchyme from the onset of lung development (Additional file 1: Figures S3 and S4). Double immunofluorescence analysis showed that GFP^+^, i.e. lineage positive cells did not contribute to the respiratory epithelium at all analyzed stages (Additional file 1: Figure S5). Quantification of GFP expression within the pulmonary mesenchyme at different developmental stages showed that *Tbx2* deletion did not alter the overall contribution of lineage positive cells in this tissue (Fig. 3a and b, Additional file 2: Table S1). Furthermore, GFP expression was detected in all ACTA2- and most TAGLN- positive SMCs, in a large fraction of EMCN- and KDR-positive endothelial cells and to a lower extend in the ALDH1A2- and WT1-positive mesothelium at E14.5. We found GFP expression in all S100A4-positive interstitial fibroblasts, as well as in over 85% of the POSTN-, in 87% of the PDGFRA- and in 61% of the PGDFRB-positive area at E16.5 (Fig. 3a, and c, Additional file 2: Table S1). Hence, loss of TBX2 does not affect the differentiation and lineage distribution of mesenchymal precursors initially positive for TBX2 in the developing lung.

**Fig. 4:**
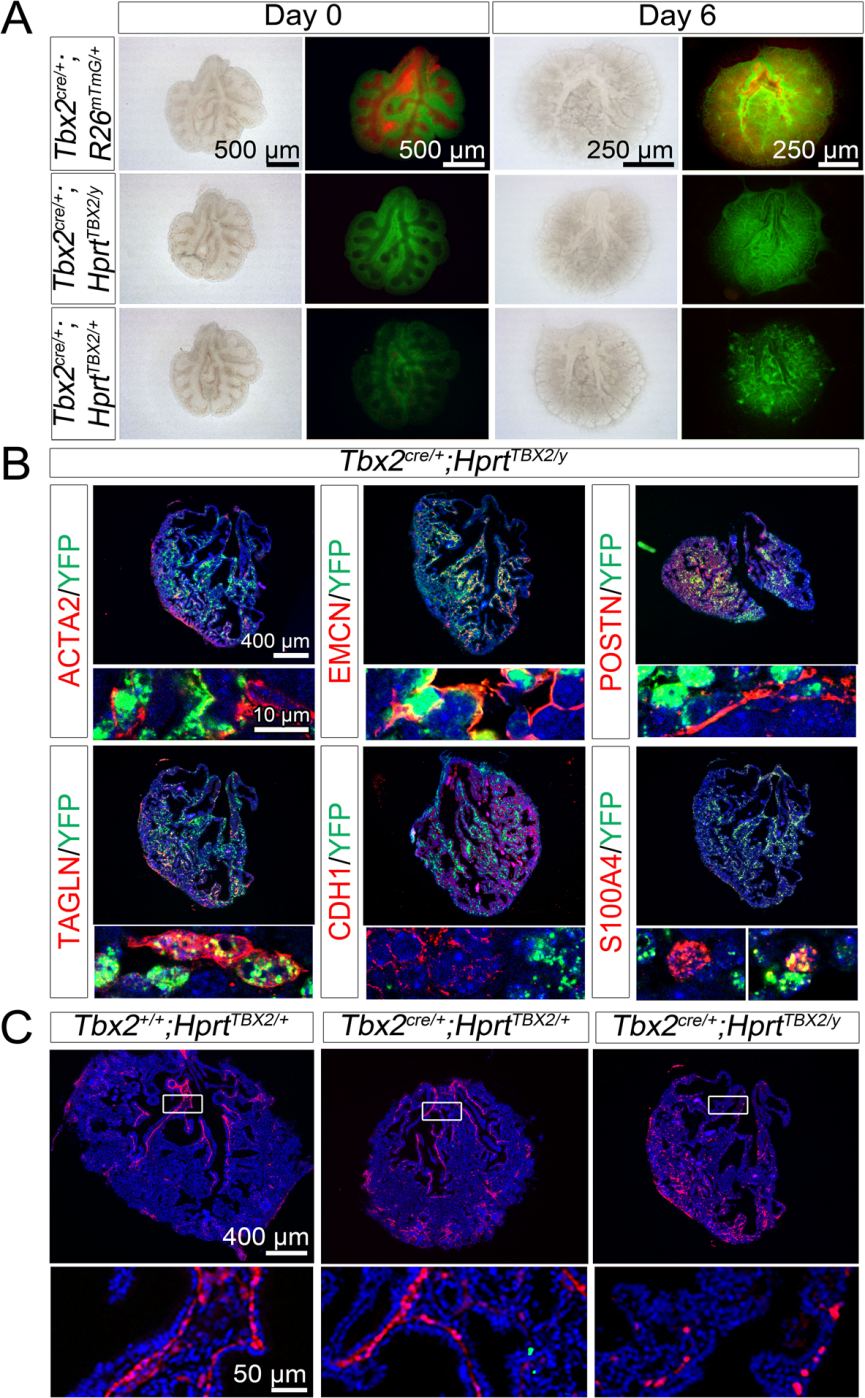
*Tbx2*-overexpression does not alter the fate of TBX2^+^ cells in the developing lung. (**a**) Morphology and GFP/RFP epifluorescence of lung explants of E12.5 *Tbx2*^*cre/+*^*;R26*^*mTmG/+*^ (control), *Tbx2*^*cre/+*^*;Hprt*^*TBX2/+*^ (female) and *Tbx2*^*cre/+*^*;Hprt*^*TBX2/y*^ (male) embryos at day 0 and day 6 of culture. (**b**) Double immunofluorescence analysis of YFP (indicating TBX2 expression from the *Hprt* allele) and cell-type specific marker proteins (TAGLN, ACTA2 for SMCs; EMCN for the endothelium; CDH1 for the epithelium; S100A4 for different types of fibroblasts and POSTN for the ECM) on sections of *Tbx2*^*cre/+*^*;Hprt*^*TBX2/y*^ lung rudiments after 8 days of culture. Antigens are color-coded. Selected regions of overview images are magnified in the row below. (**c**) Immunofluorescent analysis of TAGLN expression on frontal sections of *Tbx2*^*cre/+*^*;R26*^*mTmG/+*^ (control), *Tbx2*^*cre/+*^*;Hprt*^*TBX2/+*^ and *Tbx2*^*cre/+*^*;Hprt*^*TBX2/y*^ lung cultures. Genotypes are as indicated. Insets or selected regions in overview images are magnified in the row below. Nuclei were counterstained with DAPI

To analyze the gain-of-function situation, we used an *Hprt* knock-in allele of human *TBX2 (Hprt*^*TBX2*^) [23] which upon combination with the *Tbx2*^*cre*^ allele leads to ectopic expression in all cells of the TBX2 lineage. Due to the X-chromosomal localization of the *Hprt* locus, females exhibit mosaic overexpression, while in males all recombined cells express TBX2 ectopically. Cre-mediated recombination was visualized by co-expression of a YFP from the *Hprt*^*TBX2*^ allele. Since overexpression of TBX2 is lethal to male embryos at approximately E13.0, lung rudiments of E12.5 embryos were explanted and analyzed after culturing for 6 or 8 days (Fig. 4).

On the morphological level, explants of male *(Tbx2*^*cre/+*^*;Hprt*^*TBX2/y*^) and female *(Tbx2*^*cre/+*^*;Hprt*^*TBX2/+*^) mutant embryos did not show any obvious defects. Male explants exhibited homogenous YFP epifluorescence during the whole culture period, while explants of female mutants showed a mosaic pattern as expected (Fig. 4a). Starting from day 2 of culture, YFP^+^ cells in females formed clusters at the rim of the explants which increased with time. Similar clusters were observed in control cultures, however, they emerged approximately three days later and were unevenly distributed over the entire organ (Fig. 4a, Additional file 1: Figure S6).

We also determined TBX2 expression and lineage contribution in these cultures. In *Tbx2*^*cre/+*^*;R26*^*mTmG/+*^ control cultures both TBX2 expression as well as the TBX2^+^ cell lineage was restricted to the CDH1 negative population. The same was true for male and female overexpression mutants (Additional file 1: Figure S7). To decipher the cell-types to which the TBX2 overexpressing cells contribute in these cultures, we first validated the differentiation markers on control cultures. KDR, ALDH1A2 and WT1 were not faithfully expressed, whereas ACTA2, TAGLN, EMCN, POSTN and S100A4 were expressed in a similar fashion as in vivo (Additional file 1: Figure S8B).

In cultures of *Tbx2*^*cre/+*^*;Hprt*^*TBX2*^ embryos, TBX2^+^ cells contributed to ACTA2^+^ and TAGLN^+^ SMCs but with reduced frequency in comparison to *Tbx2*^*cre/+*^*;R26*^*mTmG/+*^ control cultures. In *Tbx2*^*cre/+*^*;Hprt*^*TBX2/y*^ cultures we observed an increase of interstitial ACTA2- and TAGLN-positive cells at the expense of SMCs lining the trachea and bronchi (Fig. 4b and c). The EMCN^+^ vasculature was composed of YFP-positive and negative cells as in the control. However, the *Tbx2*^*cre/+*^*;Hprt*^*TBX2/y*^ culture harbored clearly more EMCN^+^ cells than the female mutant or the control (Fig. 4b, Additional file 1: Figures S8, S9). Double immunofluorescence analysis for S100A4 and YFP revealed that S100A4^+^ cells partially derived from the TBX2 lineage. Similarly, YFP^+^ cells expressed POSTN both in male (Fig. 4b) and female overexpression mutants (Additional file 1: Figure S9).

To exclude that changes of TBX2 expression are compensated by opposing expression changes of the closely related TBX3 protein, we analyzed TBX3 expression in the context of the TBX2^+^ lineage both in control and in loss- and gain-of-function conditions. In the control condition, TBX3 expression was confined to the TBX2 cell lineage in the pulmonary mesenchyme at all analyzed stages of lung development (Additional file 1: Figure S10A) as well as in lung explant cultures (Addittional file 1: Figure S10B). Neither loss nor gain of TBX2 in the pulmonary mesenchyme affected TBX3 expression in this tissue (Additional file 1: Figures S10C and S10D). Together, this analysis shows that prolonged expression of TBX2 in the pulmonary mesenchyme affects the contribution of TBX2-positive cells to SMCs as well as the differentiation of SMCs and endothelial cells.

### SMC differentiation and functionality depends on TBX2

**Fig. 5:**
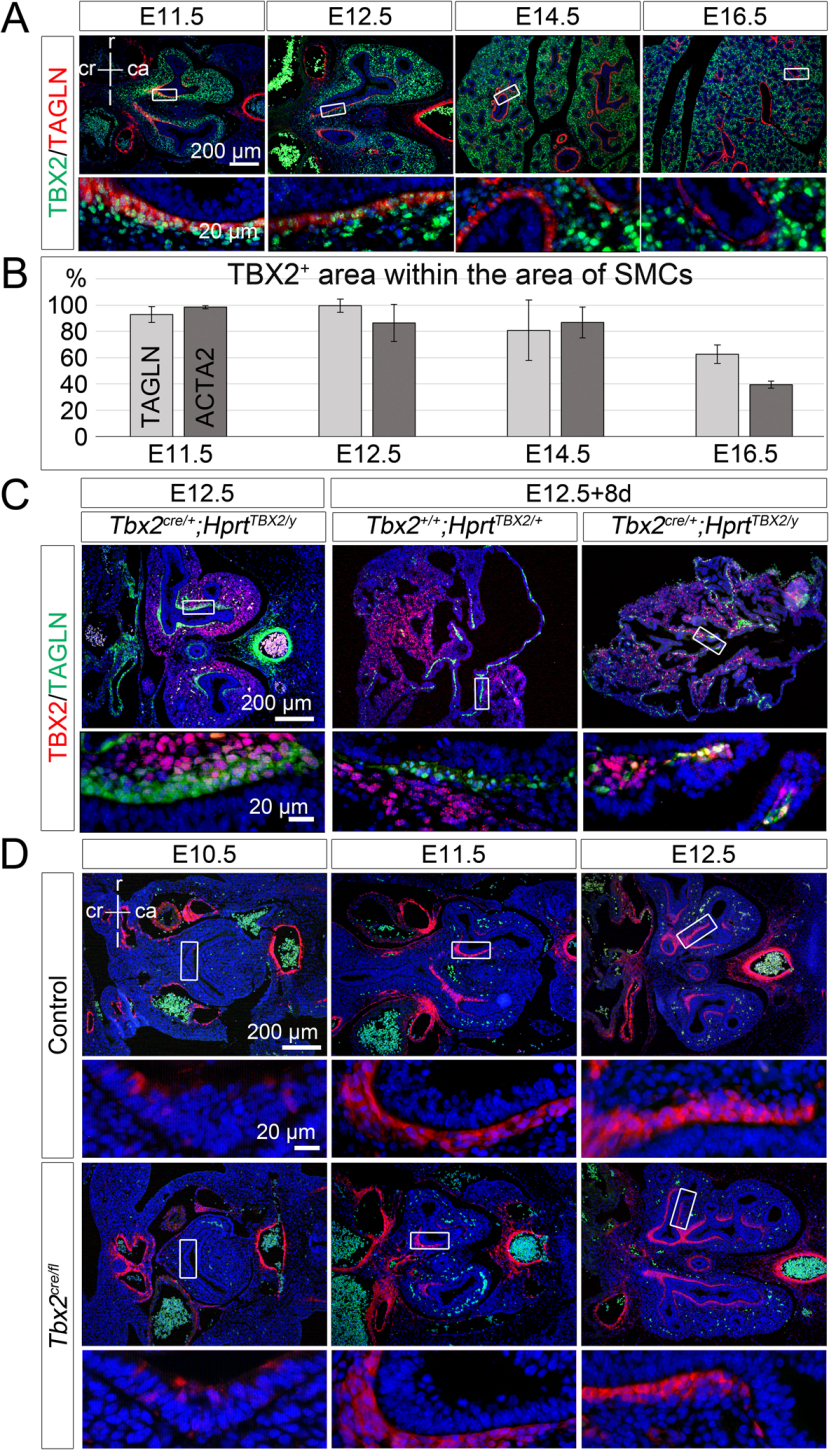
SMC development and its correlation with TBX2 expression. (**a**, **c**) Double immunofluorescence analysis of expression of TBX2 with the SMC protein TAGLN on frontal lung sections of control mice (**a**), of *Tbx2*^*cre/+*^*;Hprt*^*TBX2/y*^ lungs at E12.5, and of 8-day cultures of E12.5 *Tbx2*^*+/+*^*;Hprt*^*TBX2/+*^ and *Tbx2*^*cre/+*^*;Hprt*^*TBX2/y*^ lung explants (**c**). (**b**) Quantification of TBX2 expression in pulmonary SMCs marked by TAGLN or ACTA2 of control embryos at different embryonic stages. Average values: TAGLN^+^: 93% ± 6% (E11.5); 99% ± 5% (E12.5); 81% ± 23% (E14.5); 63% ± 3% (E16.5); ACTA2^+^: 98 ± 1% (E11.5); 87% ± 14% (E12.5); 87 ± 12% (E14.5); 39% ± 3% (E16.5). Complete data set is provided in Table S1. Technical artefacts produce values above 100%. (**d**) Immunofluorescence of TAGLN (red) on control and *Tbx2*^*cre/fl*^*;R26*^*mTmG/+*^ frontal lung sections at different developmental stages. Antigens are color-coded. Stages are as indicated. Nuclei were counter stained with DAPI. Insets of overview images are magnified in the row below. ca: caudal; cr: cranial; d: dorsal; f: foregut; l: left; lb.: lung bud; r: right; v: ventral

In our *Tbx2* overexpression mutants, we found a strongly reduced number of bronchial SMCs in cultured explants of embryonic lungs (Fig. 4b and c). To more carefully explore the relation of TBX2 expression and SMC differentiation, we analyzed the expression of TBX2 in bronchial SMCs in more detail. Immunofluorescence stainings and quantifications indicated that in control lungs expression of TBX2 was inversely correlated with that of SMC markers (Fig. 5a and b, Additional file 2: Table S1). In *Tbx2*^*cre/+*^*;Hprt*^*TBX2/y*^ lungs, bronchial SMCs were established normally at E12.5 (Fig. 5c, Additional file 1: Figure S11). After 8 days of culture, only few bronchial SMCs remained in *Tbx2*^*cre/+*^*;Hprt*^*TBX2/y*^ lung explants as mentioned before (Fig. 4b) but interestingly, some of them were still TBX2^+^, whereas TBX2 expression was excluded from SMCs in the controls (Fig. 5c, Additional file 1: Figure S11).

**Fig. 6:**
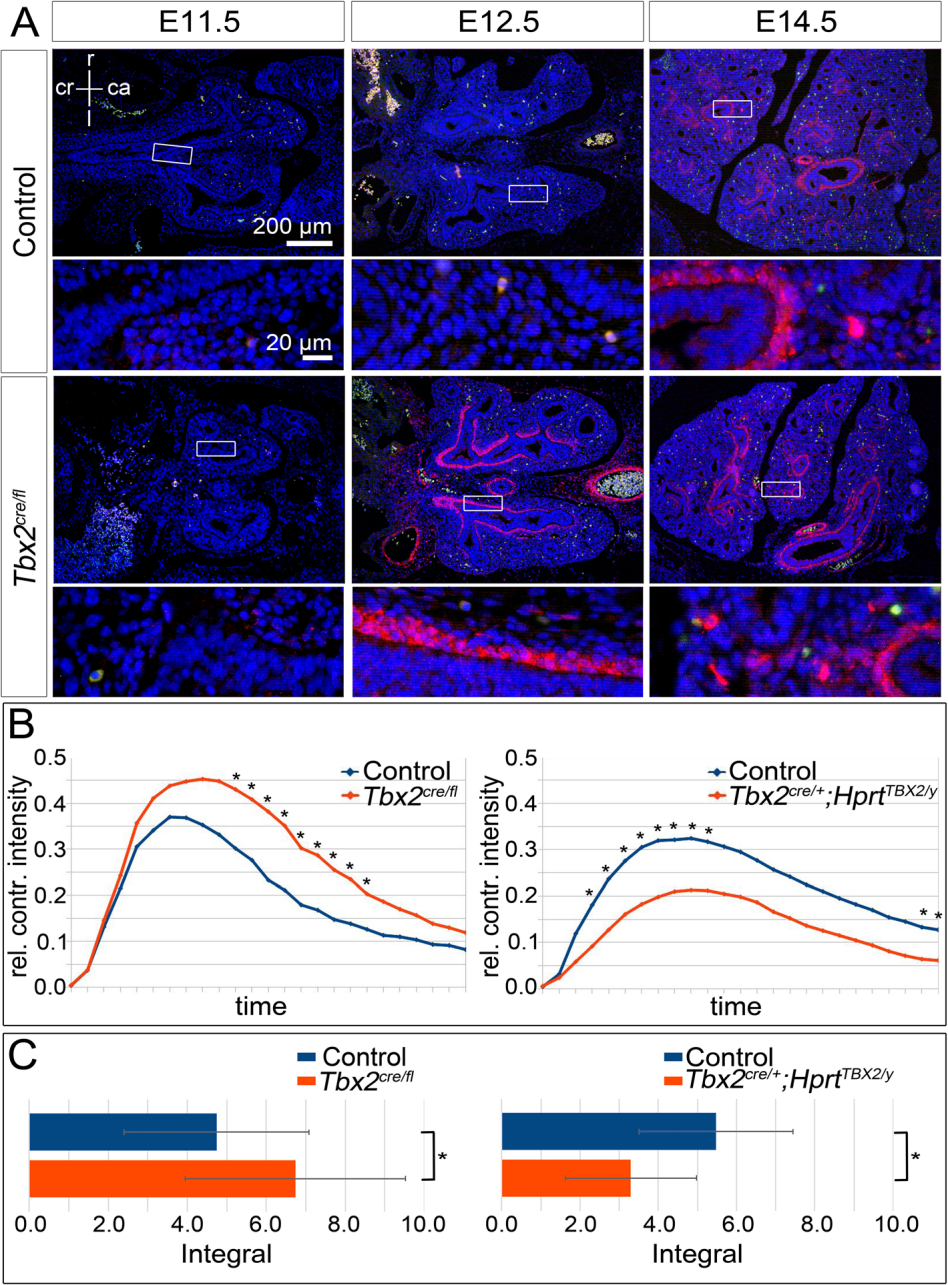
Premature S100A4 expression in TBX2-deficient bSMCs correlates with altered contraction in *Tbx2*^*cre/fl*^ and *Tbx2*^*cre/+*^*;Hprt*^*TBX2/y*^ lungs. (**a**) Immunofluorescence staining of S100A4 (red) on frontal lung section of different stages of control and *Tbx2*^*cre/fl*^*;R26*^*mTmG/+*^ embryos. Stages are as indicated. Nuclei were counterstained with DAPI. Insets of overview images are magnified in the row below. (**b**, **c**) Diagrams of relative contraction intensity (**b**) and bar graphs of corresponding integral calculation (C, Average values: Control (left): 4.7 ± 2.3, *Tbx2*^*cre/fl*^*;R26*^*mTmG*^^/+^: 6.7 ± 2.8; Control (right): 5.4 ± 2, *Tbx2*^*cre/+*^*;Hprt*^*TBX2/y*^: 3.3 ± 1.7) of the right main bronchi of lungs explanted at E12.5 and cultured for 36 h. Differences were considered significant with *p ≤ 0.05, ** *p* ≤ 0.01, *** *p* ≤ 0.005, using two-tailed Student’s t-test. Statistical values are provided in Table S2

Although *Tbx2* was not required for SMC differentiation at E14.5 (Fig. 3), its loss may affect the initiation of this program. We therefore examined expression of TAGLN (Fig. 5d) and ACTA2 (Additional file 1: Figure S12) in control and *Tbx2*-deficient lungs at E10.5, E11.5 and E12.5. However, no changes in SMC differentiation were observed at these stages. Further, the SMC related genes *myosin heavy chain 11 (Myh11), calponin1 (Cnn1)* and *desmin (Des)* showed no differential expression in *Tbx2*^*cre/fl*^ mice at E12.5 and E14.5 compared to controls (Additional file 1: Figure S12). We also analyzed expression of S100A4, which was previously described as SMC-associated Calcium-binding protein, involved in SMC function in other contexts [35]. In the control, S100A4 was first detected in bronchial SMCs at E14.5, whereas *Tbx2*^*cre/fl*^ mice showed premature expression of this protein at E12.5 (Fig. 6a).

To find out whether changes of TBX2 expression affect the functionality of pulmonary SMCs, we analyzed muscular contractions in lung explant cultures. *Tbx2*^*cre/fl*^ lungs cultured for 36 h showed an increase in contraction intensity to 45.3% compared to 39.3% of control cultures, associated with a significantly slower relaxation of the contracted muscles. *Tbx2*^*cre/+*^*;Hprt*^*TBX2/y*^ explants displayed a significant reduction to 21.3% contraction intensity compared to the control (32.4%) and a faster relaxation of the musculature after 36 h of culture (Fig. 6b). Calculating the areas below the curves clearly demonstrated the significant differences in both mutants compared to the control cultures in overall contraction intensities. In *Tbx2*^*cre/fl*^*;R26*^*mTmG/+*^ lungs, the average integral significantly increased from 4.7 to 6.7. In *Tbx2*^*cre/+*^*;Hprt*^*TBX2/y*^ lungs^*,*^ a significant decrease from 5.4 to 3.3 was observed (Fig. 6c, Additional file 3: Table S2). Together this suggests that TBX2 influences the differentiation and functionality of bronchial SMCs.

## Discussion

### TBX2 expression marks a multipotent mesenchymal progenitor population in the developing lung

To explore the lineage relation between TBX2 expressing cells and differentiated cell types in the fetal lung we performed genetic lineage tracing with a cre knock-in line into the *Tbx2* locus. Notably, previous work established that the *Tbx2*^*cre*^ allele is suited for this purpose, since *cre* expression faithfully reflects endogenous expression of *Tbx2* [20]. We found that most cells outside the respiratory epithelium, including SMCs of the airways and the vasculature, fibroblasts, a large part of endothelial cells as well as mesothelial cells of the visceral pleura were positive for the reporter. Together with the fact that TBX2 expression does not occur in the respiratory epithelium, it confirms other studies that the lineages of the respiratory epithelium and of all surrounding cell-types are completely segregated from onset of lung development ([32–34]). It also implies that TBX2 expression occurs in a common or in distinct progenitor pools of endothelial, mesothelial and mesenchymal cells of the embryonic lung. In fact, our expression analysis revealed that TBX2 is activated shortly after emergence of the lung buds, is predominantly and strongly expressed in undifferentiated cells that surround the distal lung buds and declines in differentiated SMCs and fibroblasts. It is noteworthy that TBX2 expression in the mesenchyme surrounding the distal lung buds was very heterogenous. Most cells expressed high levels, other expressed low levels or were negative for TBX2. It is conceivable that TBX2-negative cells get actually lost during lung development since they do not proliferate. Alternatively, expression levels of TBX2 in this population may fluctuate with all cells activating expression of TBX2 at some point. Notably, TBX2 expression in the endothelium and mesothelium was more variable and appeared scattered.

Besides *Tbx2*, the early pulmonary mesenchyme expresses the WNT ligand genes *Wnt2/2b*, the T-box transcription factor gene *Tbx4* and the fibroblast growth factor gene *Fgf10*. Lineage tracing of *Fgf10*-expressing cells, revealed that these cells in an early embryonic wave give rise to bronchial and vascular SMCs as well as lipofibroblasts, whereas during alveologenesis they contribute to lipofibroblasts and myofibroblasts only [35]. Even though *Fgf10* is already expressed very early and *Fgf10* is a critical factor for lung development [8, 36], the TBX2 lineage contributes to more cells within the pulmonary mesenchyme.

*Fgf10* is induced by TBX4, a T-box transcription factor that belongs with *Tbx2* to the same T-box subfamily [37, 38]. *Tbx4* is expressed in the embryonic lung mesenchyme from E9.25 onwards and lineage tracing using a *Tbx4* lung enhancer *Cre* line showed that TBX4-expressing cells give rise to a subset of fibroblasts (lipofibroblasts and myofibroblasts), SMCs, endothelial and mesothelial cells in the fetal and adult lung [38, 39]. Given the similarities of the TBX4 and the TBX2 lineages and expression patterns in the developing pulmonary mesenchyme, one might conclude that TBX2 similar to TBX4 is one of the factors, which defines the early lung mesenchyme. However, *Tbx2* deletion only affects branching morphogenesis around E14.5, i.e. much later than TBX4 [38, 40].

Previous work revealed that *Wnt2* is expressed in the ventral region of the mesenchyme surrounding the lung buds, and that these cells are able to generate most of the mesoderm/mesenchymal lineages within the lung, including bronchial and vascular SMCs, fibroblasts and proximal endothelium [7]. *Wnt2*^*+*^ cells also generate cardiomyocytes and endocardial cells within the inflow tract of the heart, demonstrating the existence of a common cardiopulmonary progenitor (CPP) that orchestrates pulmonary and cardiac development [33, 34]. Given the well-known fact that *Tbx2* is expressed early in the heart anlage and that TBX2^+^ cells also generate cardiomyocytes [20], it seems possible that TBX2 expression similar to *Wnt2* marks the CPP.

Our study observed that approximately half of pleural cells expressed TBX2 and descended from that lineage, respectively. Whether this mesothelial TBX2 progenitor is identical with the CPP or whether it represents an uncharacterized expression of TBX2 in the coelomic epithelium from which the mesothelium derives, remains unclear at this point. However, our analysis points to a molecular heterogeneity within the pleura previously not appreciated. We conclude that TBX2 expression marks an early progenitor for mesenchymal, endothelial and mesothelial cells in the lung.

### TBX2 plays a minor role in differentiation of the pulmonary mesenchyme

We have previously shown that TBX2 is required to maintain the proliferation in the lung mesenchyme by two independent molecular mechanisms: maintenance of WNT signaling and repression of cell-cycle inhibitor genes [18, 19]. In this study we found that TBX2 expression is strongly reduced upon differentiation of bronchial SMCs. Since proliferation and differentiation are often inversely correlated, we expected to see premature expression of TBX2-derived cell types, particularly SMCs and fibroblasts. However, we did not detect changes of bronchial SMCs or fibroblasts nor did we see altered lineage segregation in our loss-of-function mutants. A possible explanation is redundancy with TBX3 as in many other organ contexts [23, 41]. TBX3 is expressed in an overlapping pattern with TBX2 from E9.5 to E14.5 and then diminishes in the pulmonary mesenchyme. Combined systemic deletion of *Tbx2* and *Tbx3* leads to early embryonic death due to cardiovascular defects. In explant cultures of rare surviving double mutants the lung was severely hypoplastic and branching morphogenesis stopped very early [18, 19].

Prolonged expression of TBX2 maintained mesenchymal proliferation [18] but did not affect lineage segregation and differentiation potential of TBX2^+^ cells since SMCs, the endothelium, the mesothelium as well as fibroblasts were all found as descendants in *Tbx2*^*cre/+*^*;Hprt*^*TBX2/y*^ lungs. However, *Tbx2*^*cre/+*^*;Hprt*^*TBX2/y*^ mutants harbored strikingly more EMCN-positive cells than the female overexpression mutant or the control. Conceivably, TBX2 is sufficient to maintain the proliferative precursor population of the endothelium or ectopically induces endothelial proliferation. This is in line with our observation that TBX2 is expressed in endothelial progenitors but is less strongly expressed in endothelial cells.

We also observed in our *Tbx2* gain-of-function cultures that bronchial SMCs were established in a very small number but that interstitial or peripheral SMCs were greatly increased. Together with the observation that onset of SMC differentiation is not altered in these mutants this argues that down-regulation of TBX2 is not required for the commitment into the SMC fate but for the correct spatial allocation of these cells at the proximal airways.

Our data also suggest that TBX2 expression levels are critical for the correct physiology of SMCs in the lung. In *Tbx2* loss-of-function mutants the contraction intensity was increased whereas it was decreased in gain-of-function lung cultures. We did not find changes of major SMC structural proteins but a premature activation of S100A4 in (prospective) bronchial SMCs. Further, S100A4 is transiently expressed in bronchial SMC of controls from E14.5 to E16.5, matching the time-point when TBX2 gets reduced in this cell layer. Unfortunately, we were not able to analyze whether S100A4 expression is delayed or even completely abolished in *Tbx2*^*cre/+*^*;Hprt*^*TBX2/y*^ mice, since they do not survive until E14.5 or later, and the in vivo expression pattern is not completely reflected in culture conditions. Studies in the coronary system showed, that S100A4 promotes proliferation and migration of SMCs and that it is associated with SMC contractility [42]. Hence, repression of S100A4 by TBX2 may be one means in which this transcription factor modulates SMC physiology.

## Conclusions

Our work shows that TBX2 is expressed in an early pulmonary progenitor pool and supports a role of TBX2 in maintaining the precursor state in the pulmonary mesenchyme. The fate of pulmonary mesenchymal progenitors is largely independent of TBX2. Nevertheless, a successive and precisely timed downregulation of TBX2 is necessary to allow proper differentiation and functionality of bronchial smooth muscle cells and to limit endothelial differentiation.

## Supplementary information

**Additional file 1: Figure S1**. Secondary and tertiary antibodies do not exhibit unspecific binding. **Figure S2**. *Tbx2*/TBX2 expression and lineage contribution to the lung mesenchyme at E9.5. **Figure S3**. *Tbx2*/TBX2 expression and lineage contribution in the lung bud mesenchyme of *Tbx2*-deficient embryos*.* **Figure S4**. TBX2 expression is lost in the pulmonary mesenchyme of *Tbx2*^*cre/fl*^*;R26*^*mTmG/+*^ embryos in early lung development. **Figure S5**. The TBX2^+^ lineage does not contribute to the pulmonary epithelium in *Tbx2*-deficient embryos. **Figure S6**. Overexpression of TBX2 leads to enhanced and premature formation of lineage positive cell clusters. **Figure S7**. TBX2 expression and TBX2 lineage contribution in control and constitutively TBX2 overexpressing lung explant cultures. **Figure S8**. Validation of cell-type specific markers and of TBX2^+^ cell lineage contribution in lung explant cultures. **Figure S9**. Mesenchymal mosaic overexpression of TBX2 does not affect the lineage diversification of TBX2-expressing cells. **Figure S10**. Expression analysis of TBX3 and TBX2^+^ cell lineage contribution to TBX3 expressing cells. **Figure S11**. Analysis of ACTA2 expression in *Tbx2*^*cre/+*^*;Hprt*^*TBX2/y*^ lungs. **Figure S12**. Analysis of SMC differentiation in *Tbx2*^*cre/fl*^*;R26*^*mTmG/+*^ lungs.

**Additional file 2: Table S1.** Quantification of immunofluorescence staining. Raw measuring data and calculations of immunofluorescence signals. Quantification of TBX2 and GFP expression in the whole lung and in areas of cell type specific marker expression at different embryonic stages analyzed in this study for control and *Tbx2*^*cre/fl*^*;R26*^*mTmG/+*^ mice.

**Additional file 3: Table S2.** Statistical evaluation of contraction behavior of lung explant cultures. Raw measuring data, integral calculations and counted branching endpoints are depicted in “LOF data” and “GOF data” for controls and corresponding mutants. Statistical calculations are summarized in “LOF statistics” and “GOF statistics”.

## Data Availability

All datasets and reagents are available from the corresponding author on reasonable request.

## Abbreviations

Acta2: Actin, alpha 2, smooth muscle, aorta, (synonym: SMaA)
Aldh1a2: Aldehyde dehydrogenase family 1, subfamily A2
bSMCs: Bronchial smooth muscle cells
ca: Caudal
Cdh1: Cadherin 1
Cdkn: Cyclin-dependent kinase inhibitor
Cnn1: Calponin 1
CPP: Cardio-pulmonary precursor
cr: Cranial
d: Dorsal
DAPI: 4′,6-diamidino-2-phenylindole
Des: Desmin
E: Embryonic day
Emcn: Endomucin
f: Foregut
FCS: Fetal calf serum
Fgf10: Fibroblast growth factor 10
Frzb: Frizzled-related protein
GFP: Green fluorescent protein
GOF: Gain-of-function
Hprt: Hypoxanthine guanine phosphoribosyl transferase
hrs: Hours
Kdr: Kinase insert domain protein receptor
l: Left
lb.: Lung bud
LOF: Loss-of-function
Myh11: Myosin, heavy polypeptide 11, smooth muscle
PBS: Phosphate-buffered saline
Pdgfr: Platelet derived growth factor receptor
PFA: Paraformaldehyde
Postn: Periostin
r: Right
R26: Rosa26
S100A4: S100 calcium binding protein A4
Shisa3: Shisa family member 3
SMC: Smooth muscle cell
SMCs: Smooth muscle cells
TAGLN: Transgelin (synonym: SM22)
Tbx: T-box
v: Ventral
Wnt: Wingless-type MMTV integration site family
Wt1: Wilms tumor 1 homolog
YFP: Yellow fluorescent protein
