# Supplementary material for: TBX2-positive cells represent a multi-potent mesenchymal progenitor pool in the developing lung
Source: Respir Res. 2019 Dec 23;20:292. doi: 10.1186/s12931-019-1264-y (PMC6929292; doi:10.1186/s12931-019-1264-y)
Supplement: Supplementary file 1 — Additional file 1: Figure S1. Secondary and tertiary antibodies do not exhibit unspecific binding. Figure S2. Tbx2/TBX2 expression and lineage contribution to the lung mesenchyme at E9.5. Figure S3. Tbx2/TBX2 expression and lineage contribution in the lung bud mesenchyme of Tbx2-deficient embryos. Figure S4. TBX2 expression is lost in the pulmonary mesenchyme of Tbx2cre/fl;R26mTmG/+ embryos in early lung development. Figure S5. The TBX2+ lineage does not contribute to the pulmonary epithelium in Tbx2-deficient embryos. Figure S6. Overexpression of TBX2 leads to enhanced and premature formation of lineage positive cell clusters. Figure S7. TBX2 expression and TBX2 lineage contribution in control and constitutively TBX2 overexpressing lung explant cultures. Figure S8. Validation of cell-type specific markers and of TBX2+ cell lineage contribution in lung explant cultures. Figure S9. Mesenchymal mosaic overexpression of TBX2 does not affect the lineage diversification of TBX2-expressing cells. Figure S10. Expression analysis of TBX3 and TBX2+ cell lineage contribution to TBX3 expressing cells. Figure S11. Analysis of ACTA2 expression in Tbx2cre/+;HprtTBX2/y lungs. Figure S12. Analysis of SMC differentiation in Tbx2cre/fl;R26mTmG/+ lungs. [file 12931_2019_1264_MOESM1_ESM.doc]

**Additional file 1**


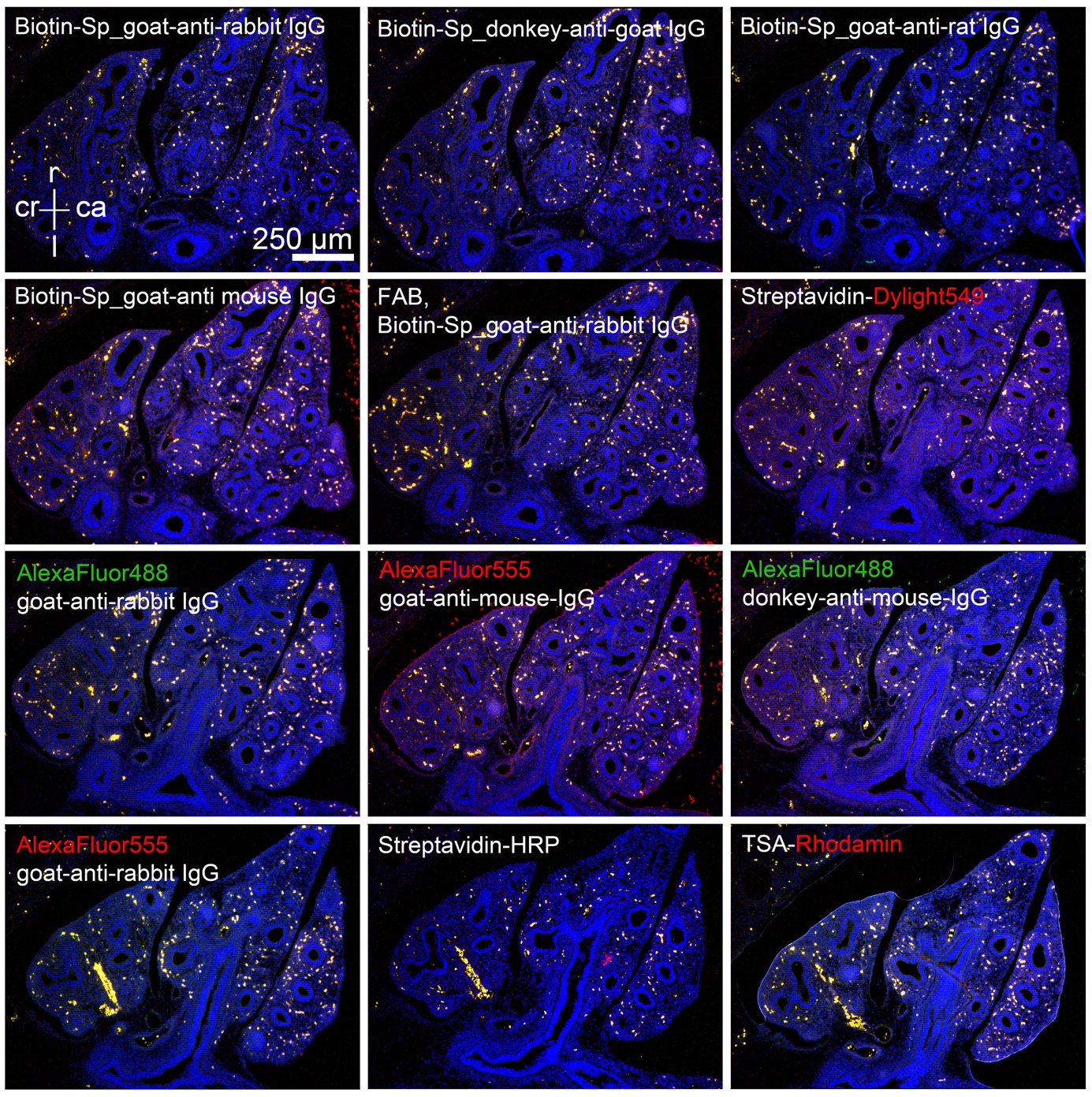


**SFigure 1. Secondary and tertiary antibodies do not exhibit unspecific binding.**

Control immunofluorescence stainings of secondary and tertiary antibodies without primary antibody on frontal lung sections of E14.5 control embryos. Antibodies and fluorophores are indicated. Incubation with a biotinylated antibody was followed by a streptavidin-HRP conjugated antibody and TSA-Rhodamine. ca: caudal; cr: cranial; l: left; r: right.

**
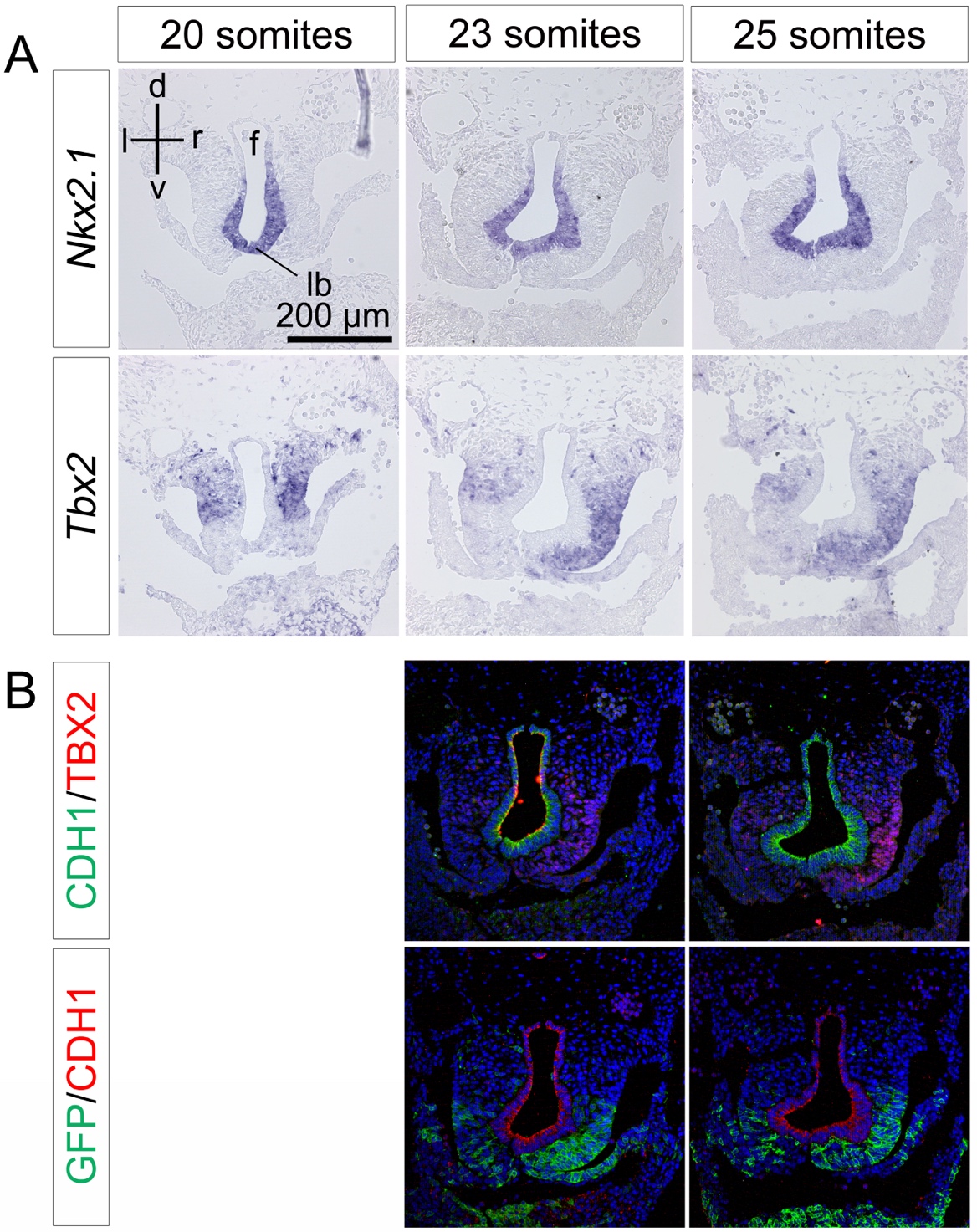
**

**SFigure 2. *Tbx2*/TBX2 expression and lineage contribution to the lung mesenchyme at E9.5.**

(A) *In situ* hybridization analysis of expression of the lung bud marker *Nkx2.1* and of *Tbx2* on adjacent transverse sections of wildtype embryos with the indicated somite numbers. (B) Double immunofluorescence analysis of expression of TBX2 with the epithelial marker CDH1 on sections of wildtype embryos, and of the lineage marker GFP with the epithelial marker CDH1 on sections of *Tbx2^cre/+^;R26^mTmG/+^* embryos. Antigens are color-coded, stages are as indicated. Nuclei were counterstained with DAPI. d: dorsal; f: foregut; l: left; lb: lung bud; r: right; v: ventral.

**
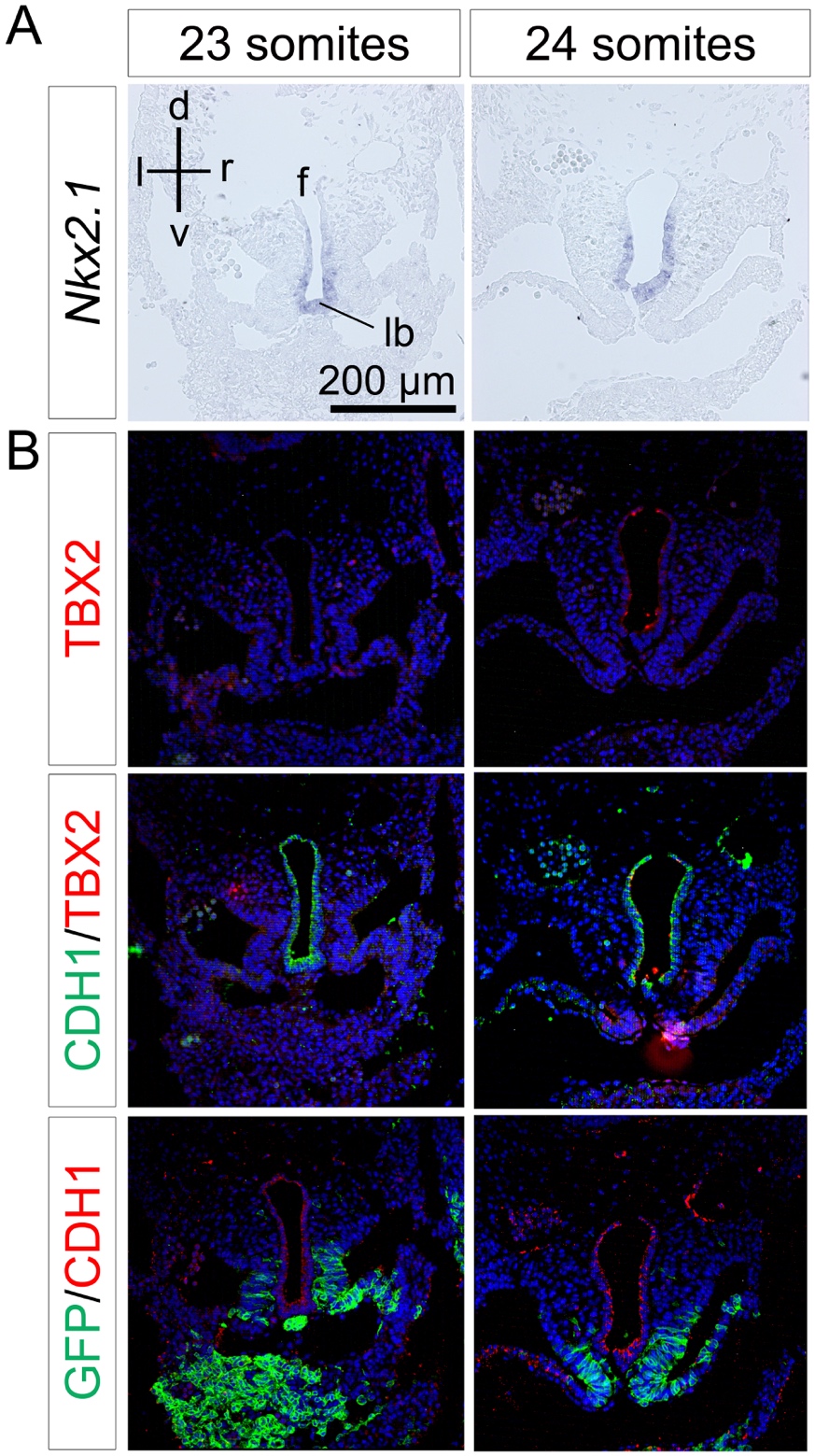
**

**SFigure 3. *Tbx2*/TBX2 expression and lineage contribution in the lung bud mesenchyme of *Tbx2*-deficient embryos.**

(A) *In situ* hybridization analysis of expression of the lung bud marker *Nkx2.1* and (B) immunofluorescence analysis of expression of TBX2 and the epithelial marker CDH1, and of the lineage marker GFP together with the epithelial marker CDH1 on transverse sections of *Tbx2^cre/fl^;R26^mTmG/+^* embryos at a developmental stage of 23 and 24 somites. Antigens are color-coded, stages are as indicated. Nuclei were counterstained with DAPI. d: dorsal; f: foregut; l: left; lb: lung bud; r: right; v: ventral.


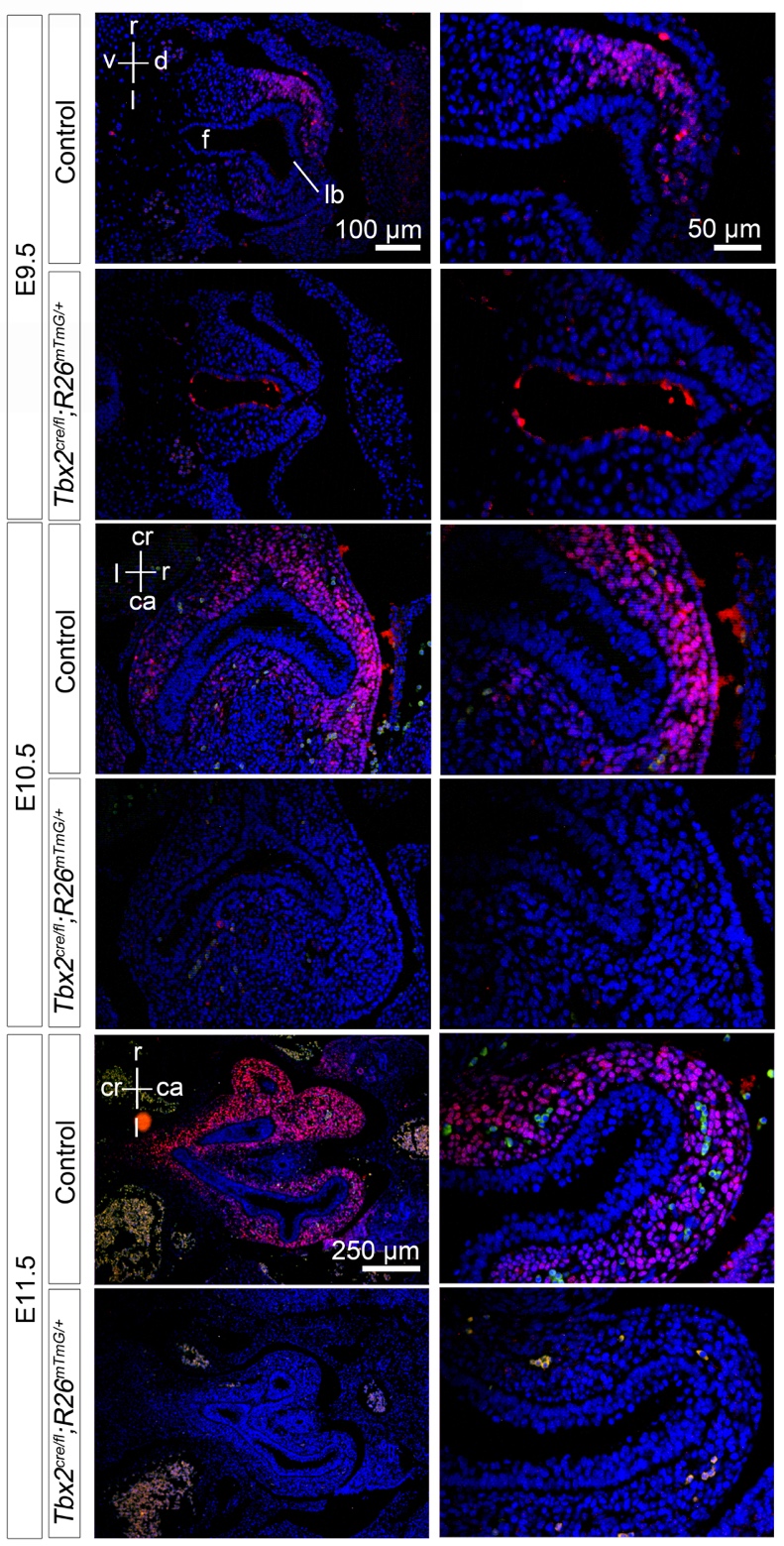


**SFigure 4. TBX2 expression is lost in the pulmonary mesenchyme of *Tbx2^cre/fl^;R26^mTmG/+^*embryos in early lung development.**

Immunofluorescence staining of TBX2 expression (red) on transverse (E9.5) and frontal (E10.5, E11.5) sections of control and *Tbx2-*deficient embryos. Higher magnifications are shown on the right panel. Stages and genotypes are as indicated. Nuclei were counterstained with DAPI. ca: caudal; cr: cranial; d: dorsal; f: foregut; l: left; lb: lung bud; r: right; v: ventral.

**
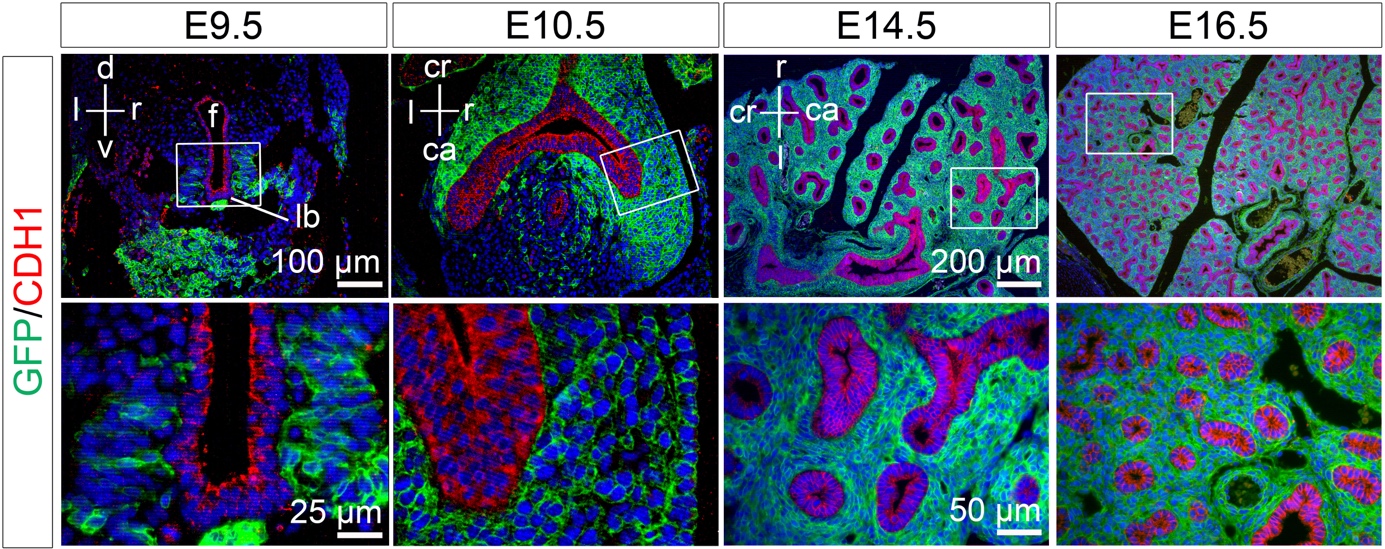
**

**SFigure 5. The TBX2^+^ lineage does not contribute to the pulmonary epithelium in *Tbx2*-deficient embryos.**

Double immunofluorescence analysis of the lineage marker GFP and the epithelial marker CDH1 on transverse (E9.5) and frontal (E10.5, E14.5, E16.5) sections of *Tbx2^cre/fl^;R26^mTmG/+^* lungs at different developmental stages. Antigens are color-coded, stages and genotypes are as indicated. Nuclei were counterstained with DAPI. Insets in overview images are magnified in the row below. ca: caudal; cr: cranial; d: dorsal; f: foregut; l: left; lb: lung bud; r: right; v: ventral.

**
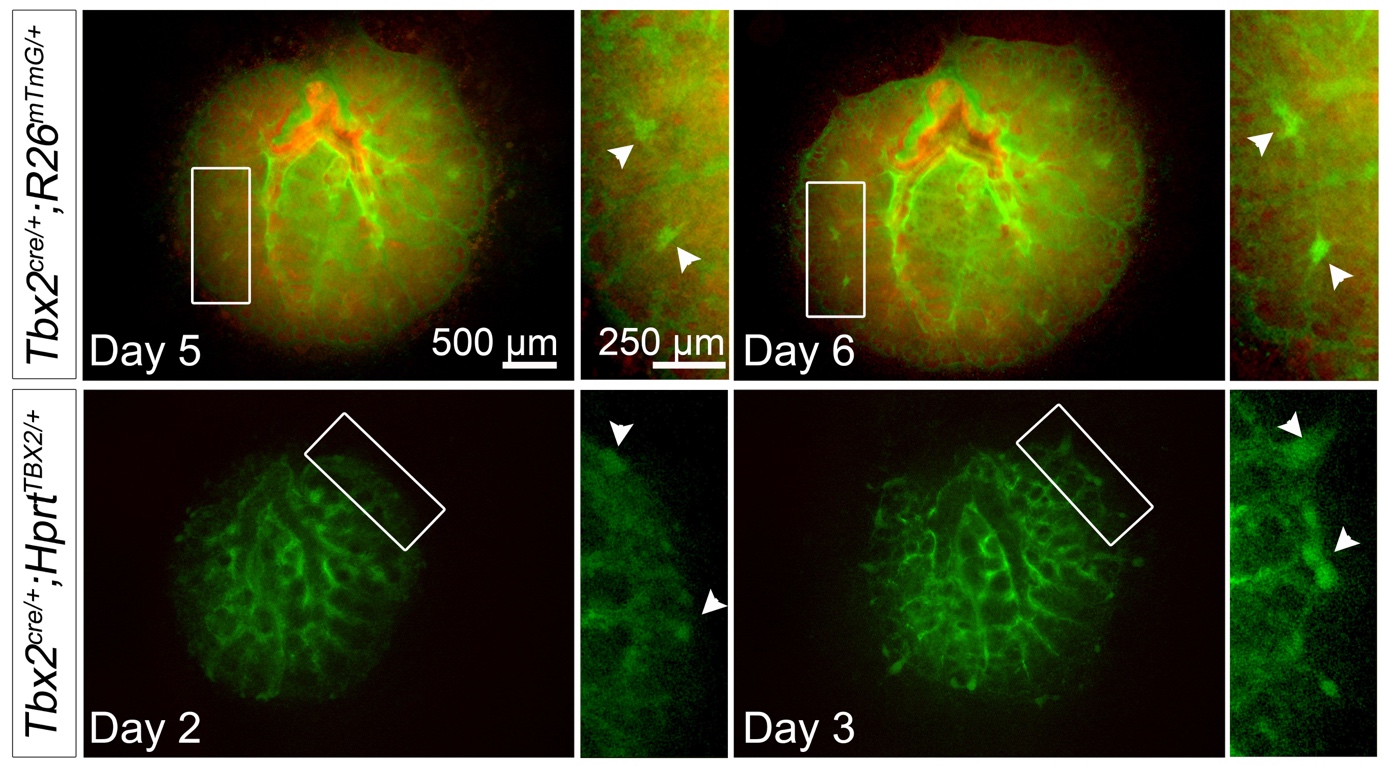
**

**SFigure 6. Overexpression of TBX2 leads to enhanced and premature formation of lineage positive cell clusters.**

Analysis of GFP/RFP epifluorescence of *Tbx2^cre/+^;R26^mTmG/+^* (control) and *Tbx2^cre/+^;Hprt^TBX2/+^* lung explants at different time-points of the culture. Clusters of irregularly distributed GFP^+^ cells (arrowheads) were observed in *Tbx2^cre/+^;R26^mTmG/+^* controls at day 5 of the culture. In *Tbx2^cre/+^;Hprt^TBX2/+^* mutant lungs GFP^+^ clusters appeared at day 2 of the culture and were evenly arranged at the rim. Stages and genotypes are as indicated. Insets in overview images are magnified on the panels on the right.

**
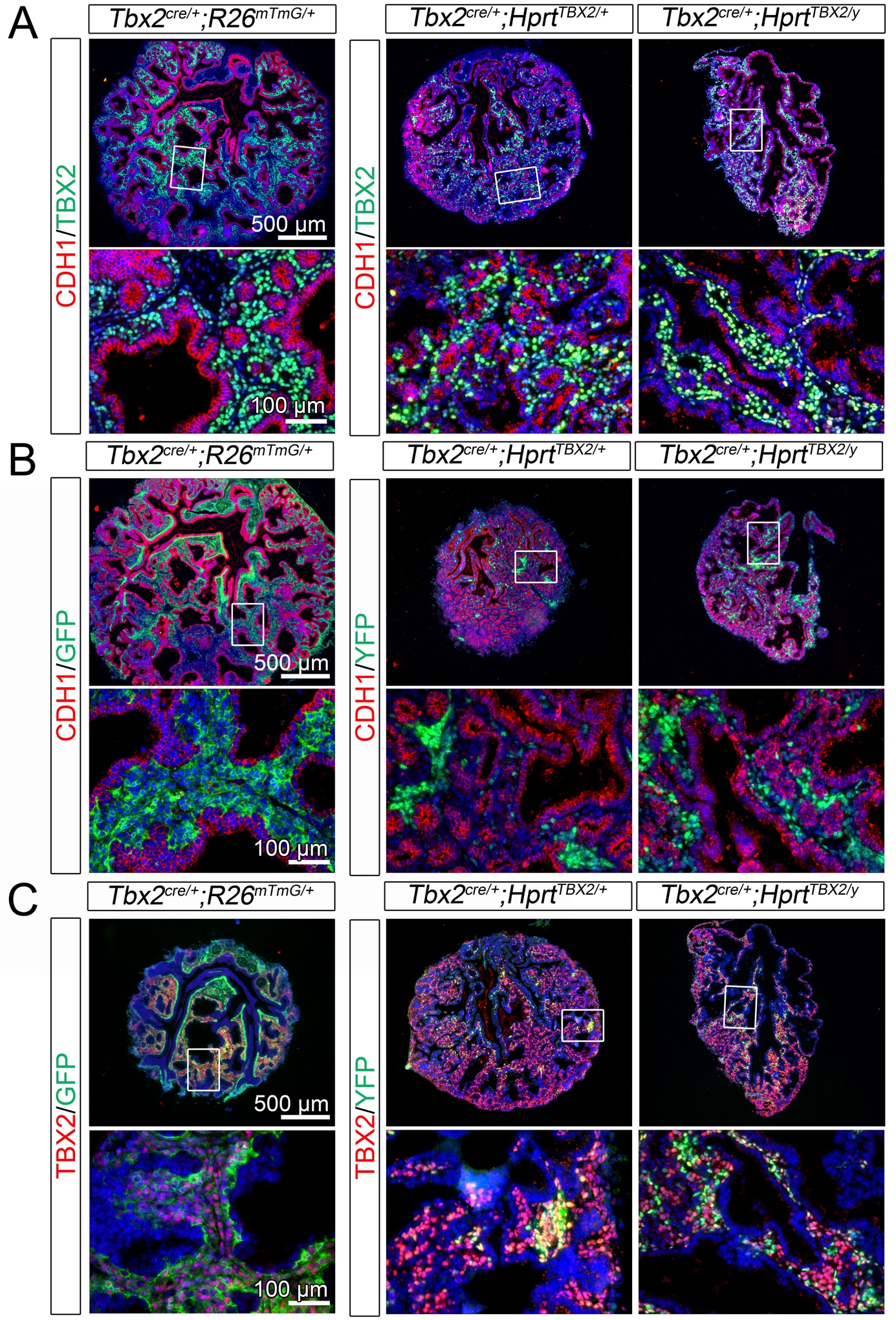
**

**SFigure 7. TBX2 expression and TBX2 lineage contribution in control and constitutively TBX2 overexpressing lung explant cultures.**

(A) Double immunofluorescence analysis of expression of TBX2 and the epithelial marker CDH1 in lung explants of *Tbx2^cre/+^;R26^mTmG/+^* (control), *Tbx2^cre/+^;Hprt^TBX2/+^* and *Tbx2^cre/+^;Hprt^TBX2/y^* embryos cultured for 6 or 8 days. (B) The distribution of lineage positive cells was analyzed by double immunofluorescence stainings of the epithelial marker CDH1 and the lineage marker GFP and YFP, respectively. (C) The correlation of TBX2 expression with TBX2 lineage was investigated using TBX2/GFP and TBX2/YFP co-stainings. Antigens are color-coded, genotypes are as indicated. Nuclei were counterstained with DAPI. Insets of overview images are magnified in the row below.

**
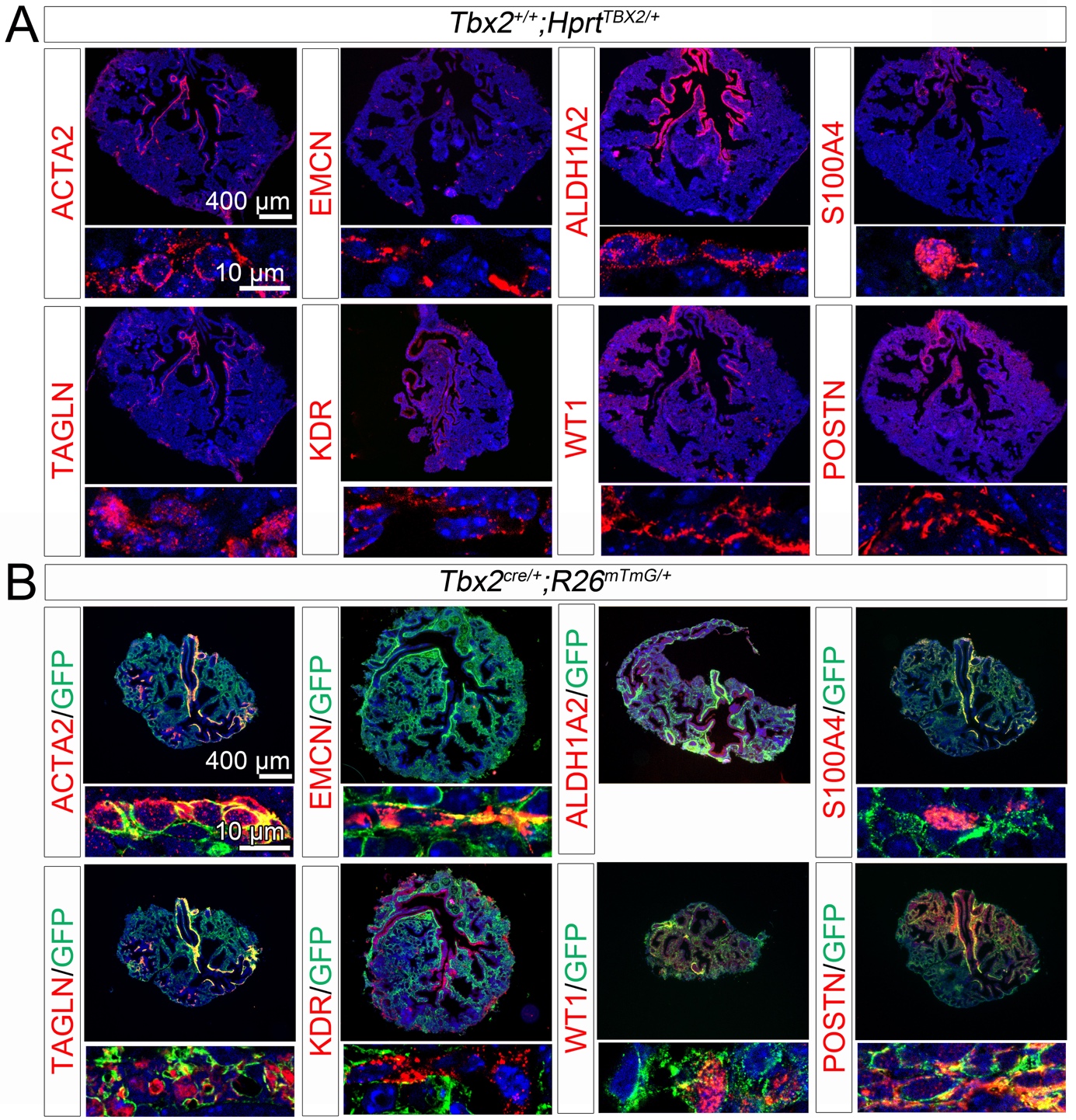
**

**SFigure 8. Validation of cell-type specific markers and of TBX2^+^ cell lineage contribution in lung explant cultures.**

(A) *Ex vivo* validation of the expression pattern of different cell-type specific markers on sections of *Cre*-negative control cultures explanted at E12.5 and cultured for 8 days. (B) Lineage tracing of TBX2-positive cells in E12.5 *Tbx2^cre/+^;R26^mTmG/+^* lung explants cultured for 6 days. Antigens are color-coded, stages and genotypes are as indicated. Nuclei were counterstained with DAPI. Selected regions of overview images are magnified in the row below.

**
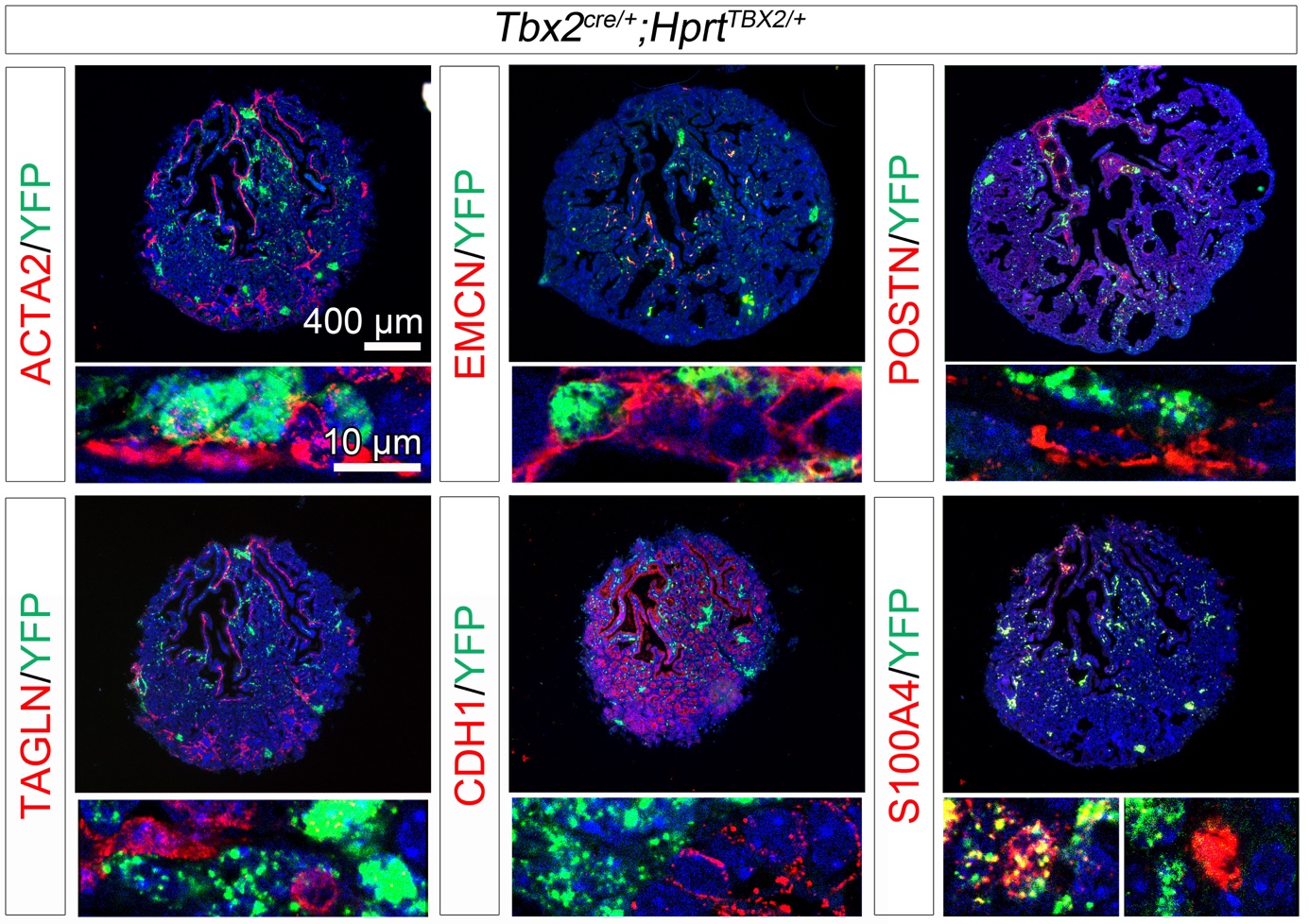
**

**SFigure 9. Mesenchymal mosaic overexpression of TBX2 does not affect the lineage diversification of TBX2-expressing cells.**

Double immunofluorescence analysis of expression of cell-type specific marker proteins (TAGLN, ACTA2 for SMCs; EMCN for the endothelium; CDH1 for the epithelium; S100A4 for different types of fibroblasts, and POSTN for the ECM) and of the TBX2 lineage marker YFP on frontal sections of explants of E12.5 *Tbx2^cre/+^;Hprt^TBX2/+^* lungs cultured for 8 days. Antigens are color-coded. Nuclei were counterstained with DAPI. Selected regions of overview images are magnified in the row below.

**
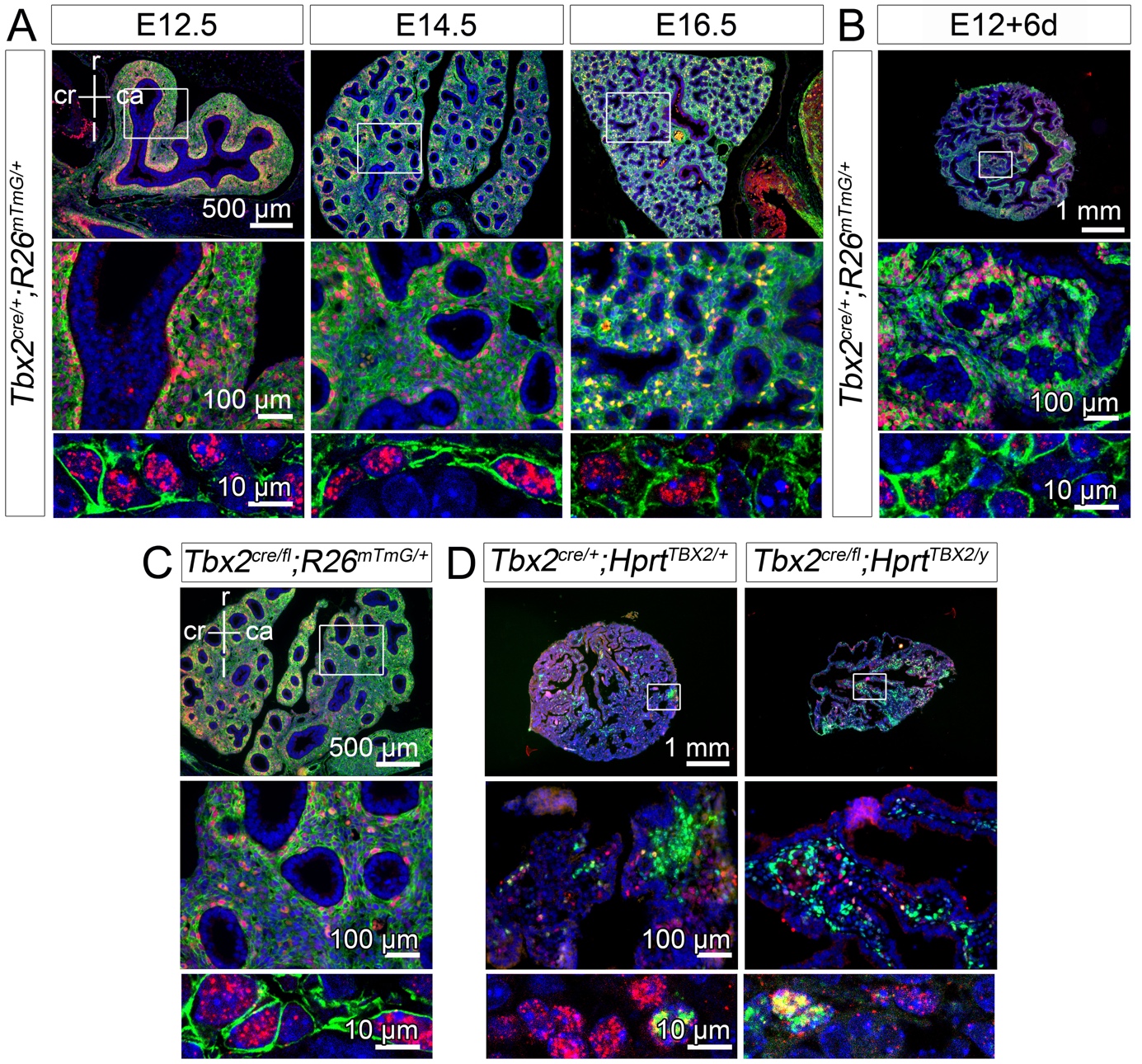
**

**SFigure 10. Expression analysis of TBX3 and TBX2^+^ cell lineage contribution to TBX3 expressing cells.**

(A, B, C) Co-immunofluorescence analysis of expression of TBX3 (in red) and the lineage marker GFP (in green) on frontal sections of lungs from *Tbx2^cre/+^;R26^mTmG/+^* control embryos at E12.5, E14.5, E16.5 (A), in 6-day cultures of E12.5 lung explants (B), and on lungs with conditional loss of *Tbx2* (*Tbx2^cre/fl^;R26^mTmG/+^*) at E14.5 (C). (D) Co-immunofluorescence analysis of expression of TBX3 (in red) and the lineage marker YFP (in green) on sections of E12.5 lung explants from *Tbx2^cre/+^;Hprt^TBX2/+^* and *Tbx2^cre/+^;Hprt^TBX2/y^* mutant embryos cultured for 8 days. Stages and genotypes are as indicated. Nuclei were counterstained with DAPI. Insets or selected regions of overview images are magnified in the rows below. ca: caudal; cr: cranial; l: left; r: right.


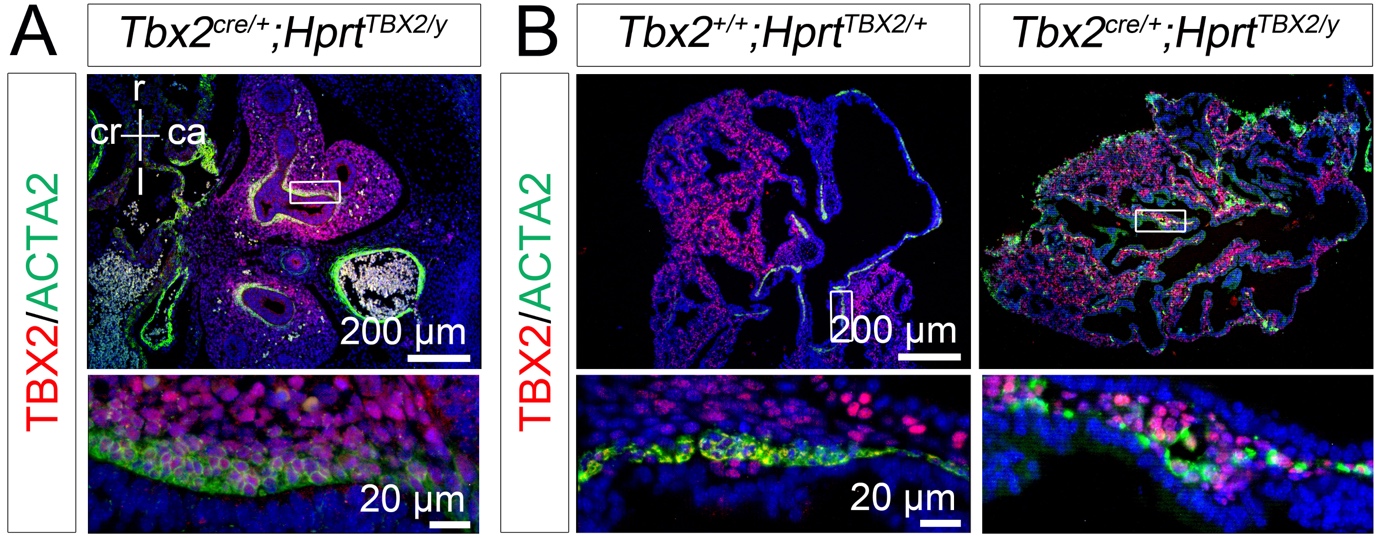


**SFigure 11. Analysis of ACTA2 expression in *Tbx2^cre/+^;Hprt^TBX2/y^* lungs.**

Double immunofluorescence analysis of expression of TBX2 and the SMC marker ACTA2 on frontal sections of E12.5 embryos (A) and on 8-day cultures of E12.5 lung explants (B). Antigens are color-coded, stages and genotypes are as indicated. Nuclei were counterstained with DAPI. Insets of overview images are magnified in the rows below. ca: caudal; cr: cranial; l: left; r: right.

**
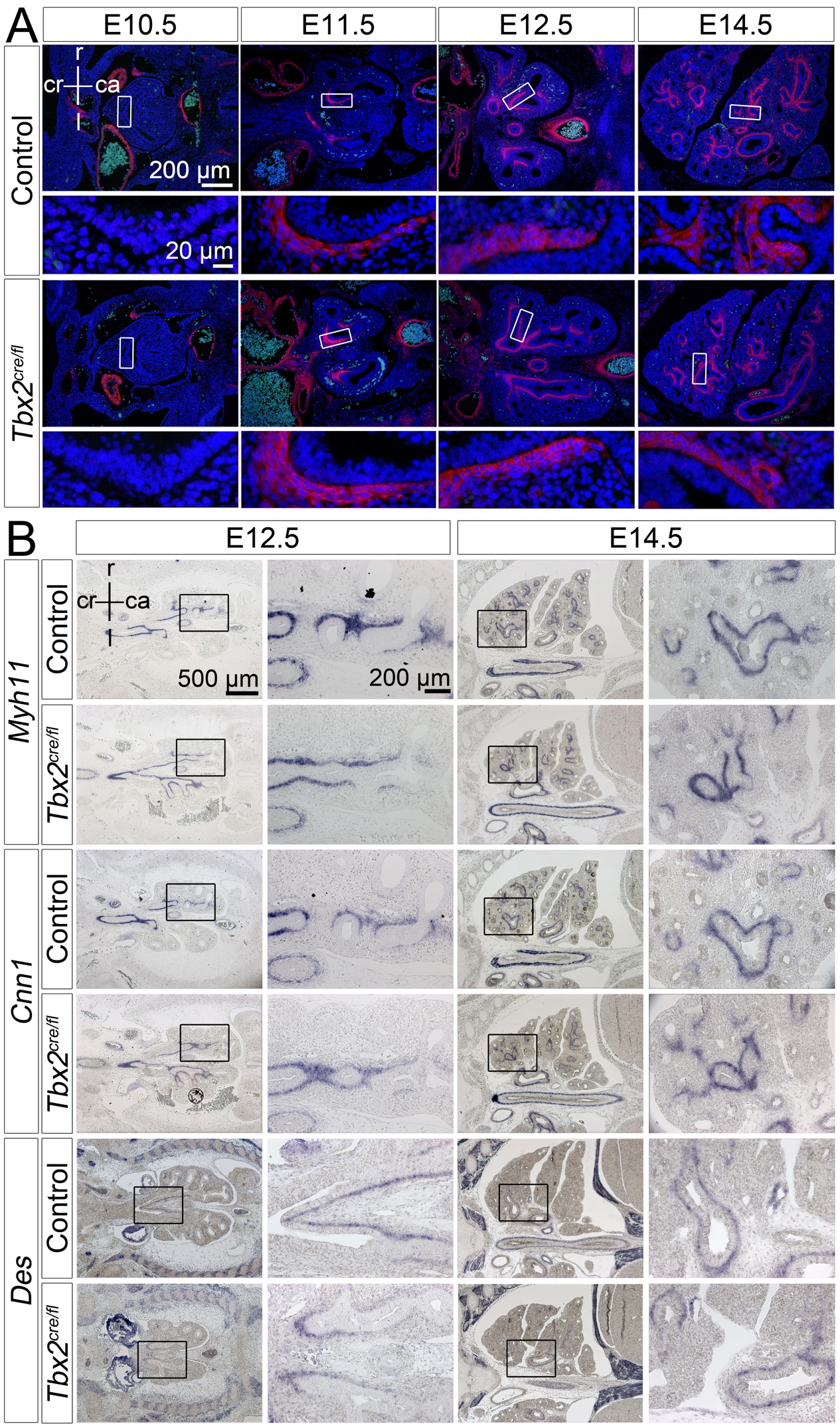
**

**SFigure 12. Analysis of SMC differentiation in *Tbx2^cre/fl^;R26^mTmG/+^* lungs.**

(A) Immunofluorescence analysis of ACTA2 expression on frontal sections of the lung of control and *Tbx2^cre/fl^;R26^mTmG/+^* mice at E10.5, E11.5, E12.5 and E14.5. Nuclei were counterstained with DAPI. (B) *In situ* hybridization analysis of expression of the SMC marker genes *Myh11, Cnn1* and *Des* on frontal lung sections of *Tbx2-*deficient and control embryos at E12.5 and E14.5. Probes, stages and genotypes are as indicated. Insets of overview images are magnified in the row below (A) or in the column to the right (B). ca: caudal; cr: cranial; l: left; r: right.
